# Acute effects of virtual reality exercise bike games on psychophysiological outcomes in college North-African adolescents with cerebral palsy: A randomized clinical trial

**DOI:** 10.12688/f1000research.143189.1

**Published:** 2023-12-15

**Authors:** Makrem Soudani, Faical Farhat, Amine Ghram, Helmi Ben Saad, Mehdi Chlif

**Affiliations:** 1 Research Laboratory Education, Motricity, Sport and Health LR19JS01, University of Sfax, Sfax, Sfax, 3000, Tunisia; 2 Cardiac Rehabilitation, Hamad Medical Corporation, Doha, Doha, 3050, Qatar; 3 Research laboratory “Heart failure, LR12SP09”, Farhat HACHED Hospital,, University of Sousse, Sousse, Sousse, Tunisia; 4 Exercise Physiology and Rehabilitation Laboratory, University of Picardy Jules Verne, Amiens, Hauts-de-France, France

**Keywords:** Cognitive Function; Decision-Making; Health; RCT; Training; Virtual Reality

## Abstract

**Background:**

Cerebral palsy (CP) is a neurological disorder that can affect motor skills and psychophysiological well-being. Virtual Reality Exercise (VRE) has been shown to improve cognitive and physical outcomes for patients with CP. Therefore, the aim of this study was to investigate the effects of VRE on attention, vigor, and decision-making abilities in adolescents with CP.

**Methods:**

A randomized controlled trial was used. Fourteen Tunisian college adolescents (15.6 ± 0.7 years; diagnosed with CP) were randomly assigned to either the VRE group or the Traditional Exercise (TE) group. The VRE group engaged in 40 min exercise sessions using VRE bike games, while the TE group participated in TE sessions.

**Results:**

Fourteen participants (42.9 % females) were included in this analysis The results showed that VRE had a significant positive impact on attention and vigor compared to TE. Participants in the VRE group demonstrated improved attention levels and reported higher levels of vigor following the exercise sessions.

**Conclusions:**

The findings suggest that VRE is an effective intervention for improving attention and vigor abilities in adolescents with CP. Further research is needed to confirm these findings and to investigate the long-term effects of VRE.

**Registration:**

Pan African Clinical Trial Registry (PACTR202308598603482; 31/08/2023).

## Introduction

Cerebral palsy (CP) is characterized by nonprogressive brain changes during fetal or infant development, leading to movement and posture abnormalities. ^1^ In addition to motor disorders, patients with CP often exhibit symptoms related to sensory, cognitive, communicative, perceptual, behavioral, and seizure abnormalities. ^2^ These patients are at an elevated risk of developing metabolic and cardiovascular diseases, as well as experiencing reduced cardiorespiratory endurance and muscle strength. ^3^ Furthermore, they tend to engage in lower levels of physical activity (PA), ^4^ which can contribute to negative health outcomes and premature mortality. ^5^^,^^6^ As the most common cause of physical impairment, CP significantly impacts the quality of life, leading to substantial economic and psychological burdens. ^7^

Treatment approaches for CP are diverse and tailored to the type, severity, and individual needs of the patient. ^8^ Physical therapy and rehabilitation are vital components of treatment, as they improve strength, flexibility, balance, motor development, and mobility. ^9^ Furthermore, occupational therapy, speech and language therapy, along with adaptive equipment, can effectively address physical impairments and enhance mobility. ^10^ To support emotional and behavioral challenges, psychologists and social workers can employ various strategies, such as behavior therapy, cognitive behavior therapy, strengths-based counseling, and mutual aid group work. ^11^ These comprehensive and interdisciplinary interventions aim to provide holistic care and optimize the overall well-being of patients with CP.

Physical exercise plays a crucial role in the treatment of children/adolescents with CP. ^12^ Physical exercise has significant implications for both short- and long- term health outcomes in CP, such as improved fitness levels, reduced risk factors for diseases, and fewer secondary complications. ^13^ However, patients with CP tend to engage in less habitual PA compared to their peers, leading to adverse effects on their overall health and well-being. ^14^ This lack of PA substantially increases the chances of developing obesity, cardiovascular diseases, and mental health problems. ^4^ Therefore, clinicians should actively promote and facilitate opportunities for increased habitual PA while also encouraging a reduction in sedentary behavior, aiming to optimize long-term health outcomes.

The development of technology presents a promising avenue for promoting PA and overall well-being. Interactive video games, such as video game cycling, virtual reality (VR), and mobile games, have proven effective in encouraging individuals of all age groups to engage in, and increase their PA levels. ^15^ VR, a form of digital technology that integrates sight, sound, touch, and smell to create an immersive experience, has gained significant attention. ^16^ In 2023, VR applications are being developed and utilized in various clinically based fields, including rehabilitative and behavioral medicine. ^17^^,^^18^ Notably, the introduction of commercially available VR exercise (VRE) equipment, such as the VirZoom—a VR-based exercise cycle compatible with the Oculus VR headset—has shown promise. ^19^ VirZoom exercise bike is an efficient, enjoyable, and motivating tool for PA, particularly among college students. ^20^ These findings suggest that health professionals can consider utilizing such technology to encourage PA participation across different populations. However, further extensive trials are necessary to confirm and determine the optimal utilization of this technology in promoting PA. In summary, technology including interactive video games and VR, holds potential as an effective means to promote PA and improve overall health. Future research should focus on validating and expanding upon the findings, establishing guidelines for incorporating this technology into PA interventions. Existing literature demonstrates that regular exercise significantly improves and maintains cognitive function and memory. ^21^ However, the effects of acute or single bouts of exercise have not been extensively explored. Literature provides growing evidence that acute aerobic exercise has a small but positive impact on human cognitive function, mood, and memory. ^22^^,^^23^ Despite these findings, there remains limited evidence-based research on the cognitive function of children and adolescents. ^24^^,^^25^

Therefore, the present randomized controlled trial (RCT) aimed to investigate the physiological and psychological outcomes of using the VirZoom VRE bike compared to traditional exercise (TE) bikes in North African college adolescents with CP. Given the entertaining and motivating nature of the VRE experience, we hypothesized that VR applications would lead to higher attention, memory, vigor, and decision-making abilities as the main outcomes in college adolescents with CP compared to TE. Additionally, we aimed to assess the impact of VRE on spasticity as the secondary outcome.

## Methods

### Ethical considerations

The study was conducted according to the guidelines of the Declaration of Helsinki, and approved by the local Ethics Committee of the High Institute of Nursing, University of Sfax, Tunisia (Protection Committee Approval Registration code: CPP SUD N 0388/2022), registered on the Pan African Clinical Trial Registry (identification number: PACTR202308598603482). Written informed consent was obtained from all the adolescents’ parents or legal guardians. Adolescents agreed verbally to participate in this study.

### Study design

This study is a subgroup analysis within the ongoing RCT registered with the Pan African Clinical Trial Registry (identification number: PACTR202308598603482) on August 31, 2023. The RCT was conducted following the guidelines established by the CONSORT statement. ^26^^,^^62^ Testing occurred between June and August 2023 at Sfax Association for the Care of the Physically Handicapped (Sfax, Tunisia). All procedures in the study adhered to the ethical standards of the institution and/or national research committee, as well as the 1964 Helsinki Declaration and its subsequent amendments or comparable ethical. ^27^ The study received approval from the University Institutional Review Board and the local ethics committee of the High Institute of Nursing, University of Sfax, Tunisia (Protection Committee Approval in 25 January 2022 Registration code: CPP SUD N 0388/2022). Before commencing the study, adolescents aged 14 to 16 years, along with their parents or guardians, received an explanation of the test protocol.

### Patients

A convenience sample of 14 college students were recruited from the aforementioned association. To select the participants, the study’s inclusion criteria were disclosed to physicians, pediatric physiotherapy clinics, special educators, speech therapists, sociologists from the above-cited association, and ambulant care services. Potential participants were approached through the association staff and social workers. These professionals then recruited them to participate in the study.

The eligibility criteria for participant selection were as follows: i) Adolescent aged between 14 and 16 years, ii) Medical diagnosis of spastic CP confirmed by a pediatric neurologist; iii) Motor function classified at level I or II according to the Gross Motor Function Classification System (GMFCS V), iv) PA levels below the international norm (i.e., less than one hour daily at >5 metabolic equivalents (MET)), indicating moderate or vigorous intensity, ^28^ v) Lack of regular sports participation (i.e., less than three sessions per week for 20 minutes or more), and vi) Reported issues with mobility in daily life or sports.

Exclusion criteria for participation in the study were: ***i)*** Engaging in a week of moderate to intense exercise exceeding 150 minutes per week; ***ii)*** GMFCS level III to V, ***iii)*** Behavioral issues that prevent participation in group activities or a movement disorder primarily dyskinetic or atactic in nature, ***iv)*** Recent surgery within the previous six months, ***v)*** Botulinum toxin treatment, and ***vi)*** Serial casting within the recent three months, or scheduled procedures during the intervention period.

The patients’ level of physical ability was categorized using the GMFCS V. ^29^ GMFCS level I or II indicate independent walking for more than 12 months and the ability to ambulate for at least 10 meters with or without a gait-assistance device (walker or crutches). GMFCS level III to V precludes the use of the Oculus Quest handheld controller, moderate to severe intellectual disability, uncontrollable seizures, or conditions that make physical training dangerous (e.g., hip dysplasia, cardiac arrhythmia, or mitochondrial defects).

### Randomization

Method used to generate the random allocation sequence: Simple random allocation was used to generate the random allocation sequence. This means that each participant had an equal chance of being assigned to either the intervention group or the control group.

Type of randomization; details of any restriction (such as blocking and block size): No restriction was used in this study. This means that participants were not blocked or stratified in any way.

Mechanism used to implement the random allocation sequence (such as sequentially numbered containers), describing any steps taken to conceal the sequence until interventions were assigned: Sequentially numbered envelopes were used to implement the random allocation sequence. The envelopes were sealed until interventions were assigned. The allocation sequence was generated by the researcher ( ***MS*** in the authors’ list). The latter also enrolled participants and assigned them to interventions.

### Experimental design

**Figure 1.**
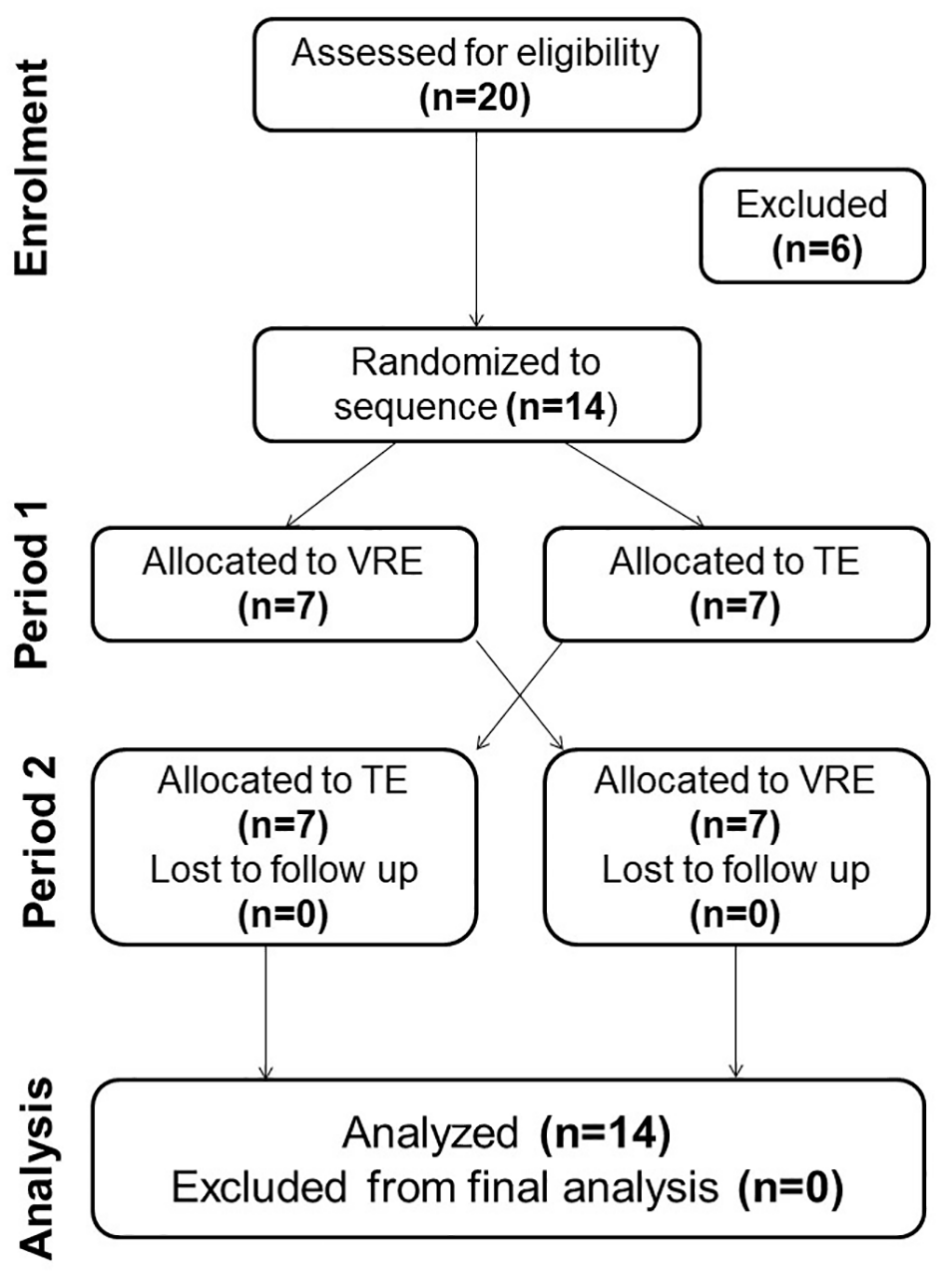
Study protocol flowchart. TE: Traditional exercise. VRE: Virtual Reality Exercise.

Figure 1 exposes the study protocol.

Anthropometric measurements, height, weight, and body mass index (BMI) were taken with patients wearing light clothing and no shoes. Patients underwent two 40-minute cycling sessions ^30^ on two distinct days. A two-day interval separated the sessions, and they were conducted in a counterbalanced manner: ***(1)*** a VirZoom VRE bike and ***(2)*** a TE bike. Both bikes are Everfit BFK-500 Kaohsiung, Taiwan. Each session consisted of three conditions. The first was a five-minute period in which the participant lay down. This was followed by a 30-minute cycling period. The third condition was another period lying down for the remaining time. The Go-No-Go task ^19^ and the Profile Of Mood States scale (POMS) ^31^ were administered two days prior to each session as well as immediately afterwards. Heart rate (bpm), blood pressure (mmHg), Modified Ashworth Scale, ^32^ and Montreal Cognitive Assessment (MoCA) ^33^ scores were recorded immediately before and after each session.

### Body composition

Height was measured with a Harpenden 602VR stadiometer to the last complete 0.1 cm, and body composition was estimated using a multifrequency bioelectrical impedance analyzer (TBF-410GS, Tanita Co., Tokyo, Japan). This is a method validated against reference methods. ^34^ The parameters collected in this study included weight, Body Mass Index, fat mass, and fat free mass.

### VirZoom VRE

VirZoom’s VZfit offers a unique experience by combining a controller attached to the handlebars and a sensor on the bike crank. When used in conjunction with an Oculus Go virtual reality headset, it allows researchers to explore various virtual destinations while the participant is stationary on a bike. ^35^

During the study, patients played two 30-minute exercise sessions on the VirZoom VZfit, which features a variety of mini-games. The games chosen for the study were “Le Tour” and “Race Car,” both of which require players to pedal faster or slower to speed up or slow down, and to lean their body side to side to turn left or right. These games were observed to be the most intense, and were played with a pedal resistance set at a medium level.

All participants were asked to maintain a moderate level of exertion, with their heart rate ranging between 65% to 85% of their Age-predicted Maximal Heart Rate (ApMHR) ^36^ as measured by a polar sensor monitor.

### Traditional stationary exercise bike

To create a training environment that was similar to traditional cycling and to reduce the environmental impact, patients in the study watched a virtual cycling video on television while using the Sparnod fitness sub-52 stationary bike. The VR cycling sessions were designed to achieve an exercise intensity equivalent to 65% to 85% of the ApMHR. ^36^

To ensure uniform exercise intensity between the traditional and VR cycling sessions, participants were instructed to maintain their heart rate between 65% and 85% of their ApMHR, which was measured by the integrated heart rate monitor on the stationary bike during the traditional cycling session.

### The Go/No-Go task

The Go/No-Go task is a response inhibition task where a motor response must either be executed or inhibited. ^37^ During this task, and using PEBL (for Psychology Experiment Building Language) computer software, participants were required to watch a sequential presentation on computer of letters and respond to a target letter by pressing a button space of the keyboard. ^38^ The presentation began with a 2 × 2 array with four stars (one in each square of the array). A single letter (P or R) was then presented in one of the squares for a duration of 500 milliseconds with an inter-stimulus interval of 1,500 milliseconds. In the first condition (P-Go), participants were asked to press a button in response to the target letter P and withhold their response to the non-target letter R. The ratio of targets to non-targets was 80:20. The first condition consisted of 160 trials. A second, reversal condition (R-Go) was then administered, and participants were now asked to make a response to the target letter R and withhold their response to the non-target letter P (the letter that they were initially conditioned to make a motor response to the task, while NoGo errors and RT to Go responses are considered as indicators of impulsivity. ^37^

The Go/No-Go task is a computerized test that evaluates inhibitory control, a crucial component of cognitive assessment and self-regulation. ^39^ During the task, participants respond by pressing a button when certain visual stimuli appear (Go trials) and withhold their response to other stimuli (No-Go trials), measuring their response inhibition. However, response inhibition can be difficult if No-Go trials are relatively rare, as Go responses become prepotent, meaning the system is biased to produce them. To ensure that inhibitory control is necessary, No-Go trials should make up less than 20% of the trials and each trial’s duration should be shorter than 1,500 ms. ^40^ To interpret data from the Go/No-Go task, precision and reaction time are measured, performance is compared with established norms, and errors of commission and omission are analyzed. Examining these factors provides insight into the participant’s response inhibition and determines whether their performance falls within the expected range. ^40^

### The Montreal Cognitive Assessment

The MoCA is a cognitive screening tool designed to detect Mild Cognitive Impairment (MCI) in the early stages of various neurodegenerative disorders. ^41^ It is a 10-minute test that is more sensitive than other commonly used screening tools, such as the Mini-Mental State Examination, making it a reliable and valid tool for detecting MCI in clinical settings. ^42^

The MoCA is composed of 30 questions that assess various cognitive domains, including orientation, executive functioning, memory, language, and visuospatial abilities. ^33^ We used for this study the Arabic version. The tasks in the MoCA include naming objects, memorizing a short list of words, drawing a clock face, and performing serial subtraction tasks. Scores on the MoCA range from 0 to 30, with a cut-off point of 26, where a score of 26 or higher is generally considered normal. ^33^

### The profile of mood state

The measurement of mood can be obtained using a valid, reliable, and sensitive self-report questionnaire called the POMS, ^43^ which consists of 58 items (i.e., words or sentences) and can provide valuable information about adolescents’ emotional states. ^44^ We used the POMS Arabic version. ^45^ The POMS is a self-report assessment tool that measures six dimensions of mood: Tension-anxiety, anger-hostility, vigor-activity, fatigue-inertia, depression-dejection and confusion-bewilderment.

The POMS is a 65-item questionnaire that is scored on a 5-point scale (0 = not at all to 4 = extremely). ^46^ It takes about 10-15 minutes to complete.

### The Physical Activity Enjoyment Scale (PACES)

The PACES is a reliable, valid and sensitive measure of PA enjoyment across populations. ^47^^,^^48^ The Arabic version of the PACES, which has been reported to be a reliable and valid measure of enjoyment of PA in Arabic-speaking populations, was applied. ^49^ The PACES consists of 18 items that are rated on a 7-point Likert scale, from 1 (I don’t enjoy it at all) to 7 (I enjoy it very much). The total score of the PACES ranges from 18 to 126, with higher scores indicating greater enjoyment of PA.

### The Modified Ashworth Scale (MAS)

The MAS assesses spasticity by measuring resistance to passive joint movement in patients with neurological disorders. ^32^ The scale assigns scores from 0 to 5 based on the degree of increased muscle tone, ranging from no increase (0) to severe increase (4). To conduct the assessment, patients are placed in a supine position, and for muscles primarily involved in flexion, the joint is moved from maximal flexion to maximal extension over one second, while muscles primarily involved in extension are moved from maximal extension to maximal flexion over one second. The investigator records the level of resistance encountered during the movement to determine the corresponding score: 0 for no resistance, 1 for minimal resistance at the end range, 1+ for a catch followed by less resistance over half the range, 2 for rigidity over more than half the range, and 3 for considerable resistance over most of the range. In this study, we have utilized the 0 to 5 version of the MAS ^50^ to measure spasticity. The latter version enables numerical quantification of spasticity severity. During the assessment, the participants were placed in a supine position, and the investigators slowly flexed and extended each joint over one second, recording the amount of resistance encountered on the scale from 0 to 5.

### Sample size and statistical analysis

A priori power analysis was conducted using G*Power3 ^51^ with the alpha level set at 0.05 and a power of 0.80, which concluded that the sample size to find significance should be 14 patients.

Values were presented as the mean ± standard deviation (SD). Cohen’s effect sizes (d) were classified as small (0.20), moderate (0.50), and large (0.80). ^52^ A 2 × 2 repeated-measures analysis of variance (ANOVA) was used to assess differences in, and between experimental trials. The two independent variables were exercise type and time. The two levels of the first independent variable (exercise type) were TE and VRE conditions. The two levels of the second independent variable (time) were pre and post-training.

Statistical analyses were performed using the IBM SPSS version 26 (IBM SPSS, Chicago, IL, USA). Alpha level was set at p<0.05.

## Results

**Figure 2.**
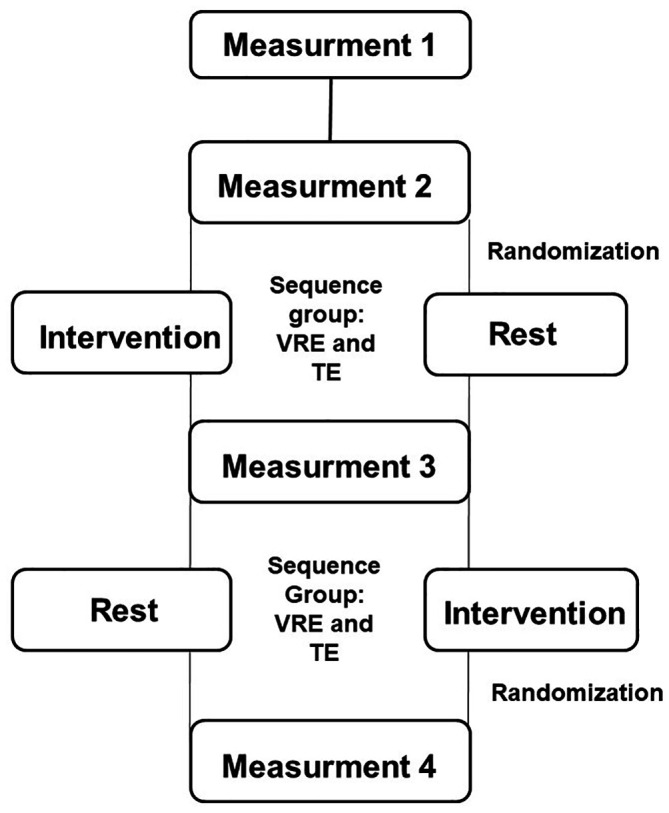
CONSORT flowchart of the recruitment process of participants into the trial. n: number. TE: Traditional exercise. VRE: Virtual Reality Exercise.

Among the initial 20 patients, only 14 were included in the final sample (7 in each group) **(** Figure 2). ^61^

Table 1 exposes the demographic characteristics of the 14 patients.

**Table 1.** Demographic characteristics of participants (n=14).

Data	Unit/category	Number or mean±Standard deviation
Sex	Boys/Girls	8/6
Traditional exercise/Virtual reality exercise		14/14
Age	Year	15.6±0.7
Height	cm	1.68± 0.11
Weight	kg	49.7±7.6
Body mass index	kg/m ^2^	18.7±3.3

Table 2 exposes the comparison of pre- and post-training scores for attention, memory, total score, vigor, decision making correct and decision making errors in both the TE and VRE training conditions.

**Table 2.** Comparison of pre- and post-training scores for attention, memory, total score, vigor, decision making correct and decision making errors in both the traditional exercise (TE) and virtual reality exercise (VRE) training conditions (n=14).

Data	TE		VRE		Statistical values ANOVA		
	Pre-training	Post-training	Pre-training	Post-training	F(1,13)	P-value	ES
Attention	1.93±0.62	3.64±1.15	1.93±0.62	4.21±1.25	6.30	0.026	0.487
Memory	2.36±0.93	4.14±0.66	2.36±0.93	3.86±0.86	3.06	0.104	- 0.231
Total Score	19.29±2.34	23.07±2.84	19.29±2.34	23.79±2.55	19.28	0.001	0.188
Vigor	14.71±3.90	14.43±7.50	14.71±3.99	18.14±2.25	2.58	0.133	1.267
Decision-making correct	286.43±21.16	282.07±19.92	290.14±16.41	299.64±11.49	26.34	0.01	0.905
Decision-making errors	33.57±21.156	37.29±20.30	29.86±16.41	21.07±13.59	28.20	0.001	-0.948

Data were mean±standard deviation. **ES**: Effect size (Cohen’s d).

### Attention

There was a significant main effect of exercise type on patients’ attention ( *F* (1,13) = 6.30, *p* = 0.026, *ηp* ^2^ = 0.327). Patients’ mean response attention was higher in VRE (SD = 1.25137) compared to TE (SD= 1.15073). This difference was a significant main effect of time F (1.13) = 97.066, p = 0.001, *ηp* ^2^ = 0.882). Patients’ mean response attention was higher at post-training (SD = 0.615) compared to pre-training (SD = 1.251). There was a statistically significant interaction between exercise type and time (F (1.13) = 6.30, *p* = 0.026, *ηp* ^2^ = 0.327).

### Memory

There was a significant main effect of exercise type on patients’ memory ( *F* (1.13) = 3.059, *p* = 0.104, *ηp* ^2^ = 0.190). Patients’ mean response memory were higher in TE (SD= 0.662) compared to VRE (SD = 0.864). This difference was a significant main effect of time F (1.13) = 180.974, p = 0.001, *ηp* ^2^ = 0.933). Patients’ mean response memory were higher at pre-training (SD = 0.928) compared to post-training (SD = 0.864). There was a statistically significant interaction between exercise type and time F (1.13) = 3.59, *p* = 0.104, *ηp* ^2^ = 0.190).

### Total score

There was a significant main effect of exercise type on patients’ total scores ( *F* (1.13) = 19.118, *p* = 0.001, *ηp* ^2^ = 0.595). Patients’ mean response total score were higher in VRE (SD = 2.547) compared to TE (SD = 2.841). This difference was a significant main effect of time F (1.13) = 80.686, p = 0.001, *ηp* ^2^ = 0.861). Descriptive statistics revealed that patients’ mean response attention was higher post-training (SD = 2.547) compared to pre-training (SD= 2.334). There was a statistically significant interaction between exercise type and time F (1.13) = 19.118, *p* = 0.001, *ηp* ^2^ = 0.595).

### Vigor

There was a significant main effect of exercise type on patients’ vigor ( *F* (1.13) = 2.576, *p* = 0.133, *ηp* ^2^ = 0.165). Patients’ mean response vigor were higher in VRE (SD =2.248) compared to TE (SD= 7.500). This difference was a significant main effect of time F (1.13) = 1.320, p = 0.271, *ηp* ^2^ = 0.092). Descriptive statistics revealed that patients’ mean response vigor were higher at post-training (SD = 2.248) compared to pre-training (SD = 3.989). There was a statistically significant interaction between exercise type and time F (1,13) = 2.576, *p* = 0.133, *ηp* ^2^ = 0.165).

### Decision-making correct

There was a significant main effect of exercise type on patients’ decision-making ( *F* (1.13) = 26.338, *p* = 0.01, *ηp* ^2^ = 0.670). Patients’ mean response decision making correct were higher in VRE (SD = 11.486) compared to TE (SD = 19.917). This difference was a significant main effect of time F (1,13) = 3.833, p = 0.72, *ηp* ^2^ = 0.228). Patients’ mean response attention were higher at post-training (SD = 11.48649) compared to pre-training (SD = 19.91700). There was a statistically significant interaction between exercise type and time F (1,13) = 27.611, *p* = 0.001, *ηp* ^2^ = 0.680).

### Decision-making errors

There was a significant main effect of exercise type on patients’ decision-making errors *F* (1,13) = 28.202, *p* = 0.001, *ηp* ^2^ = 0.684). Patients’ mean response decision-making errors were higher in TE (SD= 20.299) compared to VRE (SD = 13.589). This difference was a significant main effect of time F (1,13) = 5.350, p = 0.038, *ηp* ^2^ = 0.292). Patients’ mean response decision-making errors were higher at pre-training (SD= 21.15770) than post-training (SD= 13.58995). There was a statistically significant interaction between exercise type and time F (1,13) = 27.045, *p* = 0.001, *ηp* ^2^ = 0.675).

### MAS

Table 3 exposes the comparison of pre- and post- training scores for spasticity in both the TE and VRE training condition. Both TE and VRE interventions effectively reduced spasticity levels in various lower body regions. Notably, the VRE group exhibited more substantial improvements, with a 5.61 reduction in spasticity for the lower body (ankle + knee) compared to TE’s 3.28. In the ankle region, VRE resulted in a significant 2.29 reduction in spasticity, while TE achieved a reduction of 1.43.

**Table 3.** Comparison of pre- and post-training scores for spasticity in both the traditional exercise (TE) and virtual reality exercise (VRE) training conditions (n=14).

	TE				VRE				ES
	Pre-training	Post-training	Δ [95%CI]	Δ (%)	Pre-training	Post-training	Δ [95%CI]	Δ (%)	
Lower body (Ankle + Knee)	19.57+7.74	16.29+6.05	-3.28 [2.87, 3.69]	-16.74%	18.36+7	14.14+5.68	-5.61 [5.61, 4.11]	-25.79%	1.43
Ankle	8.79±2.74	6.5±1.95	-2.29 [1.59, 2.99]	−26.01%	7.14±2.39	5.71±2.08	-1.43 [1.37, 1.49]	20.00%	1.86
Knee	12.00±1.23	8.07±3.44	-5.74 [-13.33, 1.62]	−32.75%	11.21±5.29	7.86±3.88	-3.85 [2.73, 4.97]	-29.87%	4.79

Data were mean±standard deviation. **Δ**: Post-training minus Pre-training. **Δ (%)**: **Δ**/Pre-training. **ES**: Effect size (Cohen’s d).

## Discussion

The present study aimed to investigate the effectiveness of VRE in improving the psychophysiological outcomes of college-aged patients with CP. Our RCT demonstrated that VRE had a significant positive impact on attention, as indicated by higher scores when compared to TE. Furthermore, VRE yielded slightly higher scores in increasing patients’ vigor than TE. In terms of decision-making, a significant main effect of exercise type was observed, with VRE leading to higher scores than TE. However, it is worth noting that TE resulted in higher memory scores than VRE.

We found that VRE immediately following PA might have a more favorable impact on attention compared to TE. This finding is in line with prior research demonstrating the ability of VR to enhance cognitive function, particularly attention, and cognitive training’s potential to improve daily activities. ^53^ Additionally, this aligns with Neumann *et al*. ^54^ discovery that VR may not always be the optimal approach for diverting attention from exercise-related cues, emphasizing the importance of attentional mechanisms in shaping emotional and motivational responses to exercise in a VR environment. By directing attention towards the virtual environment, VR systems can increase the likelihood of positive impact and satisfaction, underscoring the crucial role of attentional mechanisms in shaping emotional and motivational responses during VRE. According to a 2020 research, VR environments can be customized to simulate real-life situations that require specific cognitive abilities, such as memory, decision-making, and problem-solving. ^53^ Our study investigated the acute effects of VRE on memory and compared it to TE. Our findings reveal a significant difference in memory scores between pre- and post- training, suggesting that time has a considerable impact on patients’ memory. Interestingly, the memory scores varied significantly between the TE and VRE conditions, with patients’ mean response memory being higher for TE than VRE. However, the interaction between exercise type and time was not significant, indicating that the impact of exercise type on memory did not depend on whether the memory was measured before or after the exercise. Our results differ from previous research on memory in elderly stroke survivors, which suggests that training environments can be customized to improve cognitive abilities, including memory, that influence daily activities. ^53^ Additionally, the study’s results highlight the potential benefits of immersive VR in facilitating the transfer of learned abilities from the virtual world to the real world. While some studies suggest that VRE may not be as effective as TE in enhancing memory, other studies have identified that VRE can be helpful in reducing memory decline in older adults. ^55^ Further research is necessary to confirm these findings and identify the potential advantages of exercise for memory enhancement. Therefore, the potential benefits of VRE for memory enhancement warrant further investigation.

There is evidence to suggest that there is a link between attention, memory, and vigor after immediate exercise. We report a significant main effect of exercise type on vigor, with patients reporting higher levels of vigor after VRE compared to TE. Moreover, there was a significant main effect of time on patients’ vigor, with higher levels of vigor reported after the exercise compared to before the exercise. However, there was no significant interaction between exercise type and time on patients’ vigor. Our findings are consistent with previous research indicating that acute exercise can enhance feelings of vigor in the short term. ^27^ However, our results did not align with the findings of Adhyaru *et al*., ^56^ who reported no significant difference in vigor between VRE and TE.

We identified a significant main effect of exercise type on patients’ decision-making, with higher scores observed in VRE compared to TE, suggesting that VRE may enhance decision-making abilities. However, no significant main effect of time on decision-making scores was found, indicating that the effect of exercise on decision-making did not differ significantly between pre- and post- exercise measures. Our finding diverges from previous studies that reported improvements in decision-making following acute exercise, such as Ulas *et al*. ^57^ study that found a single bout of moderate-intensity exercise improved decision-making performance in healthy adults. Our result is consistent with Shaw *et al*. ^58^ study, which reported that the use of a competitive virtual trainer while riding at a moderate-vigorous intensity did not improve cognitive performance. Conversely, our findings align with those of Ertoy Karagol *et al*. ^59^ who reported that VRE led to a significant improvement in vigor compared to the control group, as well as a significant increase in PA compared to the control group. These results suggest that VRE may be an effective intervention for improving vigor and PA in adolescents.

Furthermore, the interaction effect between exercise type and time indicated that the style of exercise and the measurement time had different effects on decision-making abilities. Thus, further investigation, such as post-hoc tests, is necessary to identify conditions that significantly varied from one another.

Our study identified significant differences in decision-making errors based on exercise type and time, indicating both have an impact. Patients made more errors during TE vs. VRE, suggesting VR provides greater cognitive benefits for decision-making. Moreover, patients made fewer errors post-training, showing exercise overall reduces decision-making errors. The significant interaction between exercise type and time demonstrated the effect of exercise on errors differed pre- and post- exercise. Benefits of VRE on errors seemed more pronounced post-training, while benefits of TE were less evident. Overall, these findings suggest that VRE may be more beneficial than TE for improving decision-making accuracy and that the effects of exercise on decision-making may vary depending on the type of exercise and timing of assessment. Our findings that VRE may be more advantageous than normal exercise for enhancing decision-making accuracy is supported previous study. ^60^ Mestre *et al*. ^60^ compared indoor cycling exercise with VR feedback vs. non-VR feedback and highlighted that cycling exercise with VR feedback had a reduced perceived exertion and an increased level of enjoyment of PA. This finding unequivocally demonstrates the beneficial effects of VR feedback and a virtual coach on the enjoyment of PA, with the virtual coach showing the greatest gains. ^60^

Our results indicate that VRE with bike has the potential to acutely reduce muscle spasticity. VRE with bike provide visual and auditory stimulation in addition to cycling, which may distract users and draw attention away from muscle sensations. ^30^ In turn, this reduction in sensory input could decrease muscle spasms and contractions, resulting in temporary relief from spasticity. ^56^ The immediate effects observed after using the VRE bike ^30^ suggest an acute impact on spasticity. However, longer-term investigations are needed to determine whether regular use of VRE with bike can yield sustained benefits for reducing spasticity. ^30^

These results emphasize the potential advantages of incorporating VRE as a promising intervention, with larger effect sizes, to enhance the physical well-being and motor skills of individuals with CP in this demographic.

### Limitations

Some potential limitations must be considered. The major limitation concerns the retrospective registration of our RCT. The main reason of the retrospective registration is the lack of experience of the first author (MS in the authors’ list). During the study execution, when he asked help from senior authors, they recommended to register the study. Since the primary purpose of trial registration is to enhance transparency, reduce bias, and promote the integrity of clinical research, a retrospective registration of an RCT can pose some challenges and may be viewed with skepticism by journal editors, peer reviewers, and readers. When an RCT is registered retrospectively, it can raise concerns about selective reporting, outcome switching, and potential bias. However, we have registered our RCT in the aim to be transparent about the timeline of the study. The registration was done as soon as possible just before the end of the trial. We have included the full study protocol with detailed information on the study design, primary and secondary outcomes, and statistical analysis plan. This demonstrate that our study was conducted in a rigorous and pre-planned manner. We confirm that our study adheres to ethical guidelines and that the retrospective registration does not compromise the ethical conduct of the trial. We have explicitly discussed how we have mitigated the risk of bias in our study, by addressing concerns related to selective outcome reporting and data-driven decisions. We attest that there was no modifications to the study design or analysis plan after data collection. Second, the study’s short duration precludes conclusions about prolonged effects. Third, the small sample size may limit generalizability. Finally, potential confounding factors such as patients’ exercise history and spasticity severity were not considered. Despite these limitations, our study has enhanced our understanding of the relationship between TEs and virtual one for adolescents with CP. We think that our findings will stimulate further investigation of this important area.

### Implications and futures directions

For adolescents with CP, VRE bike games using VirZoom’s VZfit appear to be a fun, effective alternative to boost adherence and consequently enhance mood. Future studies should be conducted in this regard to investigate additional topics related to VRE bike games for children, including assessing those with CP using objective measures to determine how long the virtual headset workouts last; assessing the interactions between individual and group activities; and determining the long-term effects of the activities performed both in the lab and at home.

## Conclusions

VRE can help improve attention, vigor, and decision-making in college students with CP more than TE. However, TE may be more beneficial for enhancing memory scores. Future research with larger samples is needed to validate our findings and identify the mechanisms driving differences in cognitive outcomes between VR and TE. Overall, both types of exercise show potential for improving mental performance and wellbeing.

## Data Availability

Zenodo: Data of 14 participants in the RCT titled Acute effects of virtual reality exercise bike games on psycho-physiological outcomes in college North-African adolescents with cerebral palsy. https://doi.org/10.5281/zenodo.10201736. ^61^ Zenodo: CONSORT checklist for: Acute effects of virtual reality exercise bike games on psycho-physiological outcomes in college North-African adolescents with cerebral palsy: A randomized Clinical trial. https://doi.org/10.5281/zenodo.10157607. ^62^ Data are available under the terms of the Creative Commons Attribution 4.0 International license (CC-BY 4.0).
