# Acute effects of virtual reality exercise bike games on psychophysiological outcomes in college North-African adolescents with cerebral palsy: A randomized clinical trial

**DOI:** 10.12688/f1000research.143189.2

**Published:** 2024-11-27

**Authors:** Makrem Soudani, Faical Farhat, Amine Ghram, Helmi Ben Saad, Mehdi Chlif

**Affiliations:** 1 Research Laboratory Education, Motricity, Sport and Health LR19JS01, University of Sfax, Sfax, Sfax, 3000, Tunisia; 2 Cardiac Rehabilitation, Hamad Medical Corporation, Doha, Doha, 3050, Qatar; 3 Research laboratory “Heart failure, LR12SP09”, Farhat HACHED Hospital,, University of Sousse, Sousse, Sousse, Tunisia; 4 Exercise Physiology and Rehabilitation Laboratory, University of Picardy Jules Verne, Amiens, Hauts-de-France, France

**Keywords:** Cognitive Function; Decision-Making; Health; RCT; Training; Virtual Reality

## Abstract

**Background:**

Cerebral palsy (CP) is a neurological disorder that can affect motor skills and psychophysiological well-being. Virtual Reality Exercise (VRE) improves cognitive and physical outcomes in patients with CP. Therefore, this study aimed to investigate the effects of VRE on attention, vigor, and decision-making abilities in adolescents with CP.

**Methods:**

A randomized controlled trial was conducted. The intervention consisted of a single 40-minute session of VRE compared to TE conducted in a controlled laboratory environment.

**Results:**

Fourteen participants (42.9 % female) were included in this analysis. The results indicated that VRE had a statistically significant positive effect on attention and vigor compared to TE. While participants in the VRE group exhibited enhanced attention levels and reported elevated levels of vigor subsequent to the exercise sessions, the memory results did not reach statistical significance.

**Conclusions:**

The findings suggest that VRE is an effective intervention for improving attention and vigor in adolescents with CP.

**Registration:**

Pan African Clinical Trial Registry (PACTR202308598603482; 31/08/2023). The trial was reported in accordance with the CONSORT reporting guidelines.

## Introduction

Cerebral palsy (CP) is a complex neurological disorder characterized by nonprogressive brain changes during fetal or infant development that lead to movement and posture abnormalities. ^1^ Beyond motor impairments, individuals with CP often experience a range of associated symptoms, including sensory, cognitive, communicative, perceptual, behavioral, and seizure abnormalities. ^2^ These challenges contribute to an increased risk of metabolic and cardiovascular diseases, reduced cardiorespiratory endurance, and diminished muscle strength. ^3^

Physical activity (PA) plays a crucial role in managing CP and improving overall health outcomes. However, individuals with CP tend to engage in lower levels of PA than their peers, ^4^ potentially exacerbating negative health outcomes and increasing the risk of premature mortality. ^5^^**,**^^6^ As the most prevalent cause of physical impairment, CP significantly influences the quality of life and poses substantial economic and psychological burdens. ^7^ Recent advancements in technology, particularly virtual reality (VR), have demonstrated promise in promoting PA and enhancing the overall well-being of various populations. VR applications have been developed and utilized in rehabilitative and behavioral medicine, ^8^^**,**^^9^ with commercially available VR exercise (VRE) equipment demonstrating potential as an efficacious and motivating tool for PA participation. ^10^

Despite extensive research on the benefits of regular exercise on cognitive function and memory, ^11^ a notable gap remains in the understanding of the effects of acute or single bouts of exercise, particularly in children and adolescents with CP. This study aimed to address this knowledge gap by investigating the physiological and psychological effects of utilizing VRE cycles compared with traditional exercise (TE) bicycles among university students with CP in North Africa.

The primary objective of this randomized controlled trial (RCT) was to examine the impact of acute exercise on cognitive function, affect, memory, and decision making in adolescents with CP. Additionally, this study explored the effects of VRE on spasticity, a crucial secondary outcome that significantly affects the quality of life of individuals with CP. By focusing on an underrepresented adolescent group and utilizing novel VR technology, this research seeks to provide valuable insights into the potential benefits of VR applications in enhancing cognitive and psychological aspects compared to conventional exercise approaches.

## Methods

### Study design

This study was a subgroup analysis of the ongoing RCT registered with the Pan African Clinical Trial Registry (identification number: PACTR202308598603482) on August 31, 2023. The RCT was conducted following the guidelines established by the CONSORT statement. ^12^ Testing occurred between June and August 2023 at Sfax Association for the Care of the Physically Handicapped (Sfax, Tunisia). All procedures in the study adhered to the ethical standards of the institution and/or national research committee as well as the 1964 Helsinki Declaration and its subsequent amendments or comparable ethical standards. ^13^ The study received approval from the University Institutional Review Board and the local ethics committee of the High Institute of Nursing, University of Sfax, Tunisia (Protection Committee Approval in 25 January 2022 Registration code: CPP SUD N 0388/2022). Before commencing the study, adolescents aged 14 to 16 years and their parents or guardians, received an explanation of the test protocol.

### Novelty of implementing the VirZoom VRE system in comparison with TE bikes, particularly within the context of adolescents with CP

Although TE bikes have been utilized in numerous rehabilitation settings, the integration of VR technology represents an innovative approach to increasing PA and improving health outcomes in this specific population. The VirZoom VRE system provides a unique and immersive fitness experience that transcends the limitations of conventional stationary cycles by offering users dynamic virtual environments to explore during exercises. This novel approach not only introduces an element of enjoyment and engagement in the training program, but also presents opportunities for cognitive stimulation and social interaction, which are particularly beneficial for adolescents with CP. Furthermore, the VirZoom VRE system enables customizable training protocols and game-based activities to address the diverse needs and abilities of individuals with CP. By incorporating games that target specific muscle groups, movement patterns, and cognitive skills, we can tailor the exercise experience to align it with the individual rehabilitation objectives and interests of participants. By emphasizing the novelty of implementing the VirZoom VRE system in this study, we underscore its potential to transform the delivery of PA interventions for adolescents with CP, ultimately leading to improved health outcomes and enhanced quality of life for this population. The selection of specific games in the VirZoom VRE system in this study was based on several rationales. Primarily, we aimed to ensure that the chosen games offered a diverse range of PAs that engaged various muscle groups and movement patterns, thus addressing the heterogeneous needs and abilities of adolescents with CP. Second, we prioritized games that are immersive, interactive, and engaging, as research has indicated that the entertainment value of VR experiences can significantly influence exercise adherence and participation. Additionally, we considered the cognitive demands of each game, selecting those that incorporated elements of attention, memory, decision-making, and problem-solving to potentially enhance cognitive outcomes in our participants. ^14^ Lastly, we aimed to incorporate games that facilitate social interaction and competition, fostering a sense of camaraderie and motivation among participants, which may further contribute to the efficacy of the intervention.

### Participants

A convenience sample of college students were recruited from the aforementioned association. To select participants, the study’s inclusion criteria were disclosed to physicians, pediatric physiotherapy clinics, special educators, speech therapists, sociologists from the above-cited association, and ambulant care services. Potential participants were approached by association staff and social workers. These professionals recruited them to participate in the study.

The eligibility criteria for participant selection were as follows: i) adolescent aged between 14 and 16 years, ii) medical diagnosis of spastic CP confirmed by a pediatric neurologist; iii) motor function classified at level I or II according to the Gross Motor Function Classification System (GMFCS V), iv) PA levels below the international norm (i.e., less than one hour daily at >5 metabolic equivalents (MET)), indicating moderate or vigorous intensity, ^15^ v) lack of regular sports participation (i.e., less than three sessions per week for 20 minutes or more), and vi) reported issues with mobility in daily life or sports.

Non-inclusion criteria for participation in the study were: ***i)*** engaging in a week of moderate to intense exercise exceeding 150 minutes per week; ***ii)*** GMFCS level III to V, ***iii)*** behavioral issues that prevent participation in group activities or a movement disorder primarily dyskinetic or atactic in nature, ***iv)*** recent surgery within the previous six months, ***v)*** botulinum toxin treatment, and ***vi)*** Serial casting within the recent three months or scheduled procedures during the intervention period.

Patients’ level of physical ability was categorized using the GMFCS V. ^16^ GMFCS level I or II indicate independent walking for more than 12 months and the ability to ambulate for at least 10 m with or without a gait-assistance device (walker or crutches). GMFCS level III to V precludes the use of the Oculus Quest handheld controller, moderate to severe intellectual disability, uncontrollable seizures, or conditions that make physical training dangerous (e.g., hip dysplasia, cardiac arrhythmia, or mitochondrial defects).

### Randomization

Simple random allocation was used to generate a random allocation sequence. This means that each participant had an equal chance of being assigned to either the VRE or TE group.

No restriction, such as blocking and block size, was applied in this study. This means that participants were not blocked or stratified.

The mechanism used to implement the random allocation sequence (such as sequentially numbered containers) described any steps taken to conceal the sequence until interventions were assigned and sequentially numbered envelopes were used to implement the random allocation sequence. The envelopes were sealed until the intervention was completed. The allocation sequence was generated by the researcher ( ***MS*** on the list of authors). The latter also enrolled participants and assigned them to interventions.

### Experimental design

**Figure 1.**
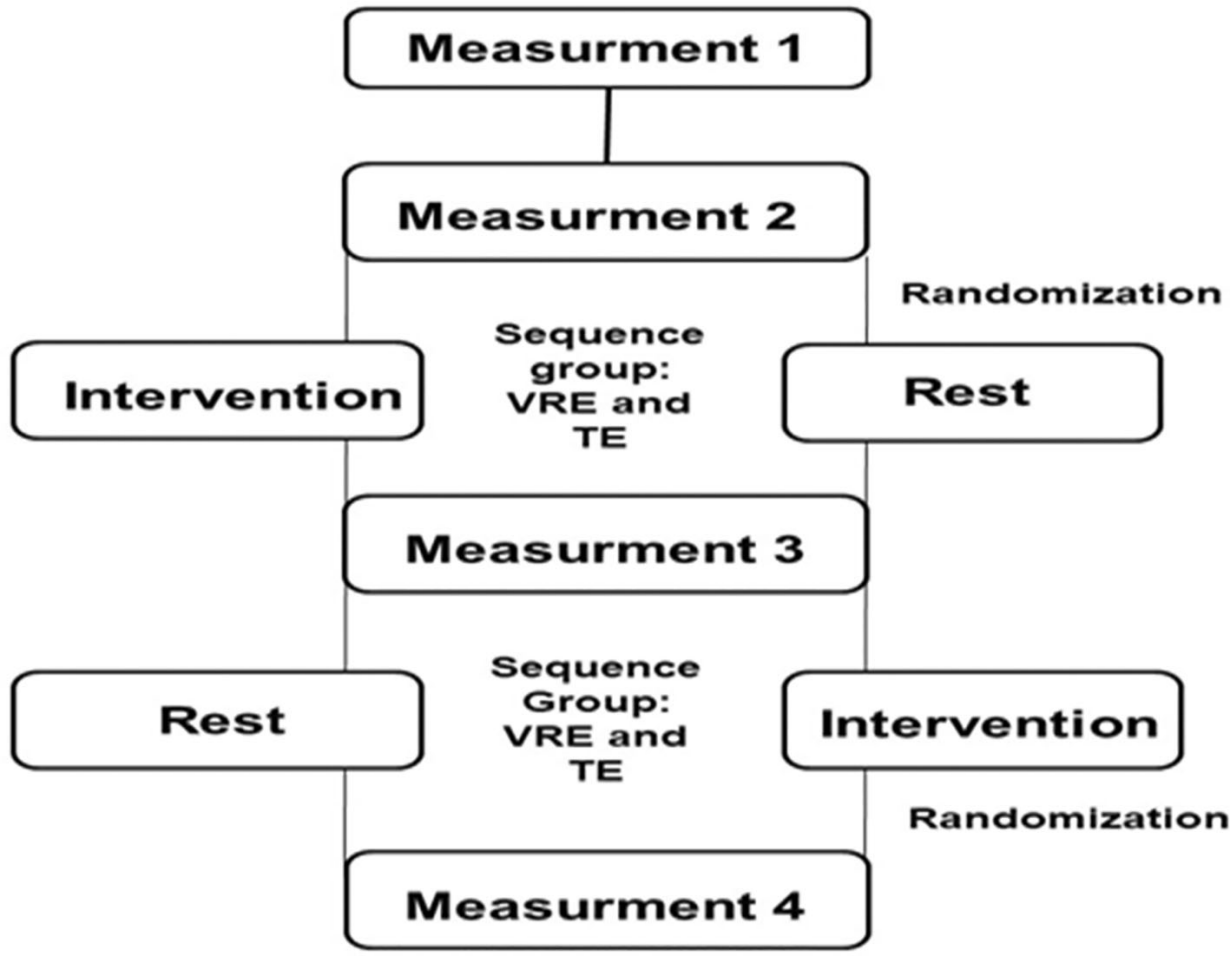
Flowchart of the study protocol. TE: Traditional exercise. VRE: Virtual reality exercise.

The study protocol is illustrated in Figure 1.

Anthropometric measurements, including height, weight, and body mass index (BMI), were recorded for the participants wearing light clothing and no shoes. The participents underwent two 40-minute cycling sessions ^17^ on two distinct days. A two-day interval separated the sessions, and they were conducted in a counterbalanced manner: ***(1)*** a VirZoom VRE bike and ***(2)*** a TE bike. Both bikes are Everfit BFK-500 Kaohsiung, Taiwan. Each session consisted of three conditions. The first was a five-minute period in which the participant lay down. This was followed by a 30-minute cycling period. The third condition was another period lying down for the remaining time. The Go-No-Go task ^9^ and the Profile Of Mood States scale (POMS) ^14^ were administered two days prior to each session as well as immediately afterwards. Heart rate (bpm), blood pressure (mmHg), Modified Ashworth Scale, ^18^ and Montreal Cognitive Assessment (MoCA) ^19^ scores were recorded immediately before and after each session.

### Body composition

Height was measured with a Harpenden 602VR stadiometer to the last complete 0.1 cm, and body composition was estimated using a multifrequency bioelectrical impedance analyzer (TBF-410GS, Tanita Co., Tokyo, Japan). This is a method validated against reference methods. ^20^ The parameters collected in this study included weight, Body Mass Index, fat mass, and fat free mass.

### VirZoom VRE

VirZoom’s VZfit offers a unique experience by combining a controller attached to handlebars and a sensor on the bike crank. When used in conjunction with an Oculus Go virtual reality headset, it allows researchers to explore various virtual destinations while the participant is stationary on a bike. ^21^

During the study, participants played two 30-minute exercise sessions on the VirZoom VZfit, which features a variety of mini-games. The games chosen for the study were “Le Tour” and “Race Car,” both of which require players to pedal faster or slower to speed up or slow down, and to lean their body side to side to turn left or right. These games were observed to be the most intense and were played with a pedal resistance set at a medium level.

All participants were asked to maintain a moderate level of exertion, with their heart rate ranging between 65% to 85% of their Age-predicted Maximal Heart Rate (ApMHR) ^22^ as measured by a polar sensor monitor.

### Traditional stationary exercise bike

To create a training environment similar to traditional cycling and to reduce the environmental impact, participants watched a virtual cycling video on television while using the Sparnod fitness sub-52 stationary bike. VR cycling sessions were designed to achieve an exercise intensity equivalent to 65% to 85% of the ApMHR. ^22^

To ensure uniform exercise intensity between the traditional and VR cycling sessions, participants were instructed to maintain their heart rate between 65% and 85% of their ApMHR, which was measured by the integrated heart rate monitor on the stationary bike during the traditional cycling session.

### The Go/No-Go task

The Go/No-Go task is a response inhibition task in which a motor response must be executed or inhibited. ^23^ During this task using the Psychology Experiment Building Language (PEBL) computer software, participants button on a keyboard. ^24^ The presentation began with a 2 × 2 array with four stars (one in each square of the array). A single letter (P or R) was then presented in one of the squares for a duration of 500 milliseconds with an inter-stimulus interval of 1,500 milliseconds. In the first condition (P-Go), participants were asked to press a button in response to the target letter P and withhold their response to the non-target letter R. The ratio of targets to non-targets was 80:20. The first condition consisted of 160 trials. A second, reversal condition (R-Go) was then administered, and participants were now asked to make a response to the target letter R and withhold their response to the non-target letter P (the letter that they were initially conditioned to make a motor response to the task, while NoGo errors and RT to Go responses are considered as indicators of impulsivity. ^23^

The Go/No-Go task is a computerized test that evaluates inhibitory control, a crucial component of cognitive assessment and self-regulation. ^25^ During the task, participants respond by pressing a button when certain visual stimuli appear (Go trials) and withhold their response to other stimuli (No-Go trials), measuring their response inhibition. However, response inhibition can be difficult if No-Go trials are relatively rare, as Go responses become prepotent, meaning the system is biased to produce them. To ensure that inhibitory control is necessary, No-Go trials should make up less than 20% of the trials and each trial’s duration should be shorter than 1,500 ms. ^26^ To interpret data from the Go/No-Go task, precision and reaction time are measured, performance is compared with established norms, and errors of commission and omission are analyzed. Examining these factors provides insight into the participant’s response inhibition and determines whether their performance falls within the expected range. ^26^

### The Montreal Cognitive Assessment

The MoCA is a cognitive screening tool designed to detect Mild Cognitive Impairment (MCI) in the early stages of various neurodegenerative disorders. ^27^ It is a 10-minute test that is more sensitive than other commonly used screening tools, such as the Mini-Mental State Examination, making it a reliable and valid tool for detecting MCI in clinical settings. ^28^

The MoCA is composed of 30 questions that assess various cognitive domains, including orientation, executive functioning, memory, language, and visuospatial abilities. ^19^ We used for this study the Arabic version. The tasks in the MoCA include naming objects, memorizing a short list of words, drawing a clock face, and performing serial subtraction tasks. Scores on the MoCA range from 0 to 30, with a cut-off point of 26, where a score of 26 or higher is generally considered normal. ^19^

### The Profile of Mood State

Mood can be measured using a valid, reliable, and sensitive self-report questionnaire called the POMS, ^29^ which consists of 58 items (i.e., words or sentences) and can provide valuable information about adolescents’ emotional states. ^30^ We used the POMS Arabic version of the POMS. ^31^ The POMS is a self-report assessment tool that measures six dimensions of mood: Tension-anxiety, anger-hostility, vigor-activity, fatigue-inertia, depression-dejection and confusion-bewilderment.

The POMS is a 65-item questionnaire that is scored on a 5-point scale (0 = not at all to 4 = extremely). ^32^ It takes about 10-15 minutes to complete.

### The Physical Activity Enjoyment Scale (PACES)

The PACES is a reliable, valid and sensitive measure of PA enjoyment across populations. ^33^^,^^34^ The Arabic version of the PACES, which has been reported to be a reliable and valid measure of enjoyment of PA in Arabic-speaking populations, was applied. ^35^ The PACES consists of 18 items that are rated on a 7-point Likert scale, from 1 (I don’t enjoy it at all) to 7 (I enjoy it very much). The total score of the PACES ranges from 18 to 126, with higher scores indicating greater enjoyment of PA.

### The Modified Ashworth Scale

The MAS assesses spasticity by measuring resistance to passive joint movement in patients with neurological disorders. ^18^ The scale assigns scores from 0 to 5 based on the degree of increased muscle tone, ranging from no increase (0) to severe increase (4). To conduct the assessment, participants are placed in a supine position, and for muscles primarily involved in flexion, the joint is moved from maximal flexion to maximal extension over one second, while muscles primarily involved in extension are moved from maximal extension to maximal flexion over one second. The investigator records the level of resistance encountered during the movement to determine the corresponding score: 0 for no resistance, 1 for minimal resistance at the end range, 1+ for a catch followed by less resistance over half the range, 2 for rigidity over more than half the range, and 3 for considerable resistance over most of the range. In this study, we have utilized the 0 to 5 version of the MAS ^36^ to measure spasticity. The latter version enables numerical quantification of spasticity severity. During the assessment, the participants were placed in a supine position, and the investigators slowly flexed and extended each joint over one second, recording the amount of resistance encountered on the scale from 0 to 5.

### Sample size and statistical analysis

A priori power analysis was conducted using G*Power3 ^37^ with the alpha level set at 0.05 and a power of 0.80, which concluded that the sample size to find significance should be 14 participants.

Values were presented as the mean ± standard deviation (SD). Cohen’s effect sizes (d) were classified as small (0.20), moderate (0.50), and large (0.80). ^38^ A 2 × 2 repeated-measures analysis of variance (ANOVA) was conducted to examine the effects of exercise type (TE vs. VRE) and time (pre- vs. post- exercise) on performance scores. Data were analyzed using SPSS version 28.0. Prior to the analysis, Mauchly’s test of sphericity was performed to assess the assumption of sphericity. If Mauchly’s test was significant, indicating a violation of sphericity, the degrees of freedom were corrected using Greenhouse-Geisser or Huynh-Feldt corrections, as appropriate. ^39^ The main effects and interactions were evaluated, and post-hoc tests were conducted using the Bonferroni correction to further explore the significant effects. Descriptive statistics, including the means and SDs for each condition, were calculated to provide a comprehensive understanding of the data. Statistical significance was set at an alpha level of .05 for all analyses.

## Results

**Figure 2.**
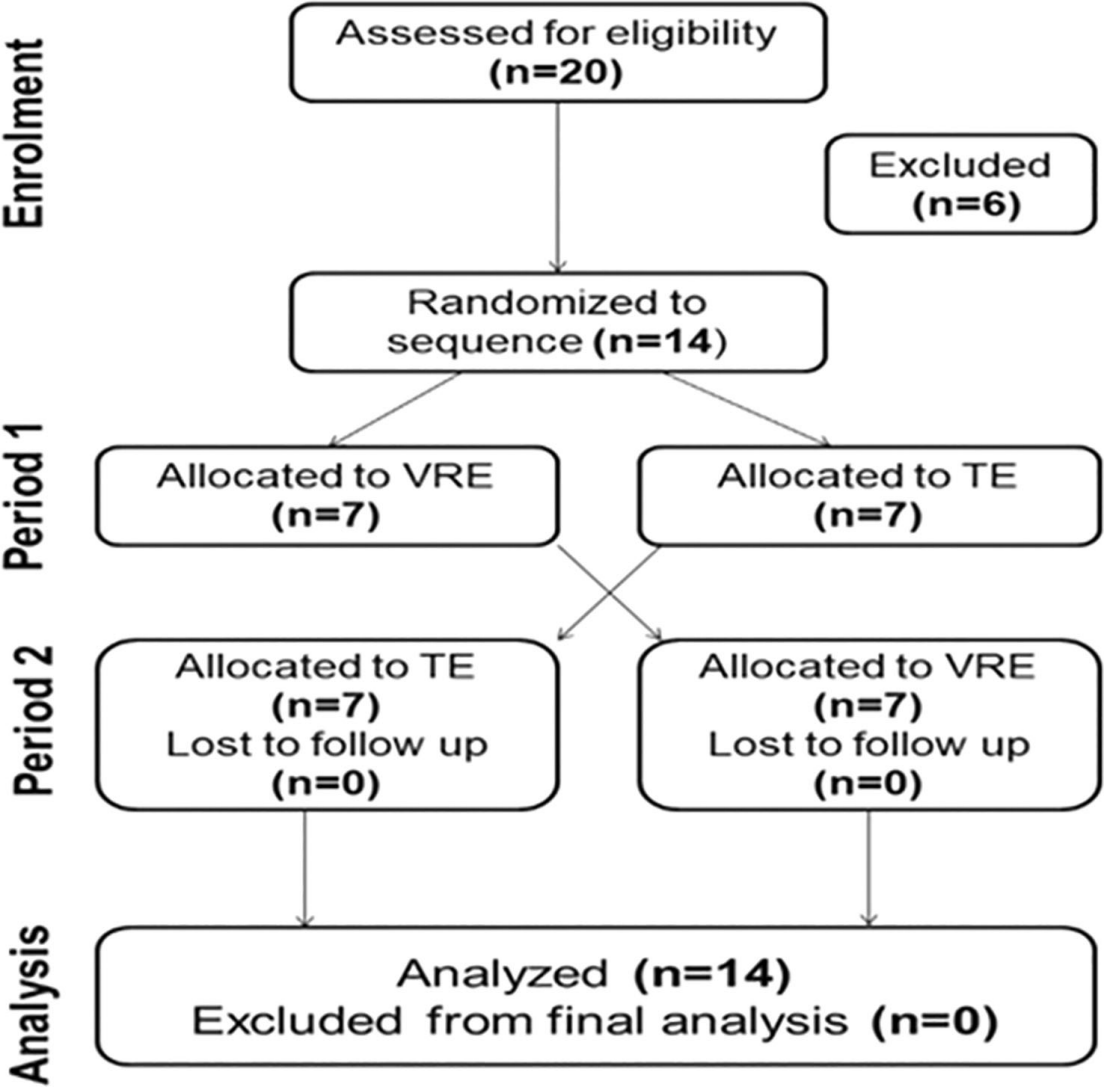
CONSORT flowchart of the recruitment process for participants in the trial. n: number. TE: Traditional exercise. VRE: Virtual reality exercise

Among the initial 20 participants, only 14 (seven in each group) were included in the final sample (7 in each group) ( Figure 2).

**Table 1.** Demographic characteristics of participants.

Characteristic	TE group (n=7)	VRE group (n=7)	Total population (n=14)
**Age (months)**	185.2 ± 9.4	188.5 ± 8.1	186.8 ± 8.8
**Sex (Boys)**	4 (57.1%)	3 (42.9%)	7 (50.0%)
**Height (cm)**	145.3 ± 12.5	147.8 ± 10.2	146.6 ± 11.4
**Weight (kg)**	45.2 ± 8.1	46.7 ± 9.4	45.9 ± 8.7
**Body mass index (kg/m** ^**2**^ **)**	21.2 ± 3.5	20.9 ± 2.7	21.1 ± 3.1

Notes: Quantitative and categorical data were mean ± standard deviation and number (%), respectively.

TE: Traditional exercise. VRE: Virtual reality exercise.

Table 2 shows the comparison of pre- and post-training scores for attention, memory, total score, vigor, correct decision making, and decision-making errors in both the TE and VRE training conditions.

**Table 2.** Comparison of pre- and post-training scores for attention, memory, total score, vigor, correct decision making, and decision-making errors in both the TE and VRE training conditions using 2 × 2 repeated-measures analysis of variance (ANOVA).

Data	TE group (n=7)		VRE group (n=7)		ANOVA		
	Pre-training	Post-training	Pre-training	Post-training	*F* (1,13)	*P* -value	ES
Attention	1.93±0.62	3.64±1.15	1.93±0.62	4.21±1.25	6.30	0.026	0.487
Memory	2.36±0.93	4.14±0.66	2.36±0.93	3.86±0.86	3.06	0.104	- 0.231
Total Score	19.29±2.34	23.07±2.84	19.29±2.34	23.79±2.55	19.28	0.001	0.188
Vigor	14.71±3.90	14.43±7.50	14.71±3.99	18.14±2.25	2.58	0.133	1.267
Decision-making correct	286.43±21.16	282.07±19.92	290.14±16.41	299.64±11.49	26.34	0.01	0.905
Decision-making errors	33.57±21.156	37.29±20.30	29.86±16.41	21.07±13.59	28.20	0.001	-0.948

Notes: Data were mean ± standard deviation.

ES: Effect size (Cohen’s d). TE: Traditional exercise. VRE: Virtual reality exercise.

### Attention

There was a significant main effect of exercise type on attention ( *F* (1,13) = 6.30, *p* = 0.026, *ηp* ^2^ = 0.327). Participants’ mean response attention was higher in VRE (SD = 1.25137) compared to TE (SD = 1.15073). This difference was a significant main effect of time *F* (1.13) = 97.066, *p* = 0.001, *ηp* ^2^ = 0.882). Participants’ mean response attention was higher at post-training (SD = 0.615) compared to pre-training (SD = 1.251). There was a statistically significant interaction between exercise type and time ( *F* (1.13) = 6.30, *p* = 0.026, *ηp* ^2^ = 0.327).

### Memory

There was a significant main effect of exercise type on the participants’ memory ( *F* (1.13) = 3.059, *p* = 0.104, *ηp* ^2^ = 0.190). The participants’ mean response memory were higher in TE (SD= 0.662) compared to VRE (SD = 0.864). This difference was a significant main effect of time *F* (1.13) = 180.974, *p* = 0.001, *ηp* ^2^ = 0.933). Participants’ mean response memory were higher at pre-training (SD = 0.928) than post-training (SD = 0.864). There was a statistically significant interaction between the exercise type and time *F* (1.13) = 3.59, *p* = 0.104, *ηp* ^2^ = 0.190).

### Total score

There was a significant main effect of exercise type on the participants’ total scores ( *F* (1.13) = 19.118, *p* = 0.001, *ηp* ^2^ = 0.595). Participants’ mean response total score were higher for VRE (SD = 2.547) than for TE (SD = 2.841). This difference was a significant main effect of time *F* (1.13) = 80.686, *p* = 0.001, *ηp* ^2^ = 0.861). Descriptive statistics revealed that participants’ mean response attention was higher post-training (SD = 2.547) than pre-training (SD = 2.334). There was a statistically significant interaction between exercise type and time *F* (1.13) = 19.118, *p* = 0.001, *ηp* ^2^ = 0.595).

### Vigor

The exercise type had no significant effect on patient vigor ( *F (*1.13) = 2.576, *p* = 0.133, *ηp* ^2^ = 0.165). Participants’ mean response vigor was higher in VRE (SD = 2.248) than in TE (SD = 7.500). This difference was a significant main effect of time ( *F* (1.13) = 1.320, *p* = 0.271, *ηp* ^2^ = 0.092). Descriptive statistics revealed that the participants’ mean response vigor was higher post-training (SD = 2.248) than pre-training (SD = 3.989). There was a statistically significant interaction between exercise type and time ( *F* (1,13) = 2.576, *p* = 0.133, *ηp* ^2^ = 0.165).

### Decision-making correct

There was a significant main effect of exercise type on participants’ decision-making ( *F* (1.13) = 26.338, *p* = 0.01, *ηp* ^2^ = 0.670). The participants’ mean response decision making correct were higher in VRE (SD = 11.486) compared to TE (SD = 19.917). This difference was a significant main effect of time *F* (1,13) = 3.833, *p* = 0.72, *ηp* ^2^ = 0.228). Participants’ mean response attention were higher at post-training (SD = 11.48649) compared to pre-training (SD = 19.91700). There was a statistically significant interaction between exercise type and time *F* (1,13) = 27.611, *p* = 0.001, *ηp* ^2^ = 0.680).

### Decision-making errors

There was a significant main effect of exercise type on the participants’ decision-making errors *F* (1,13) = 28.202, *p* = 0.001, *ηp* ^2^ = 0.684). Participants’ mean response decision-making errors were higher in TE (SD = 20.299) compared to VRE (SD = 13.589). This difference was a significant main effect of time *F* (1,13) = 5.350, *p* = 0.038, *ηp* ^2^ = 0.292). Participants’ mean response decision-making errors were higher at pre-training (SD = 21.15770) than post-training (SD = 13.58995). There was a statistically significant interaction between exercise type and time *F* (1,13) = 27.045, *p* = 0.001, *ηp* ^2^ = 0.675).

### MAS

Table 3 shows a comparison of pre- and post- training scores for spasticity in both the TE and VRE training conditions. Both TE and VRE interventions effectively reduced spasticity levels in various lower body regions. Notably, the VRE group exhibited more substantial improvements, with a 5.61 reduction in spasticity for the lower body (ankle and knee) than the TE group (3.28). In the ankle region, VRE resulted in a significant 2.29 reduction in spasticity, whereas TE reduced spasticity by 1.43.

**Table 3.** Comparison of pre- and post-training scores for spasticity in both the traditional exercise (TE) and virtual reality exercise (VRE) training conditions (n=14).

	TE group (n=7)				VRE group (n=7)				ES	*p*
	Pre-training	Post-training	Δ [95%CI]	Δ (%)	Pre-training	Post-training	Δ [95%CI]	Δ (%)		
Lower body (Ankle + Knee)	19.57+7.74	16.29+6.05	-3.28 [2.87, 3.69]	-16.74%	18.36+7	14.14+5.68	-5.61 [5.61, 4.11]	-25.79%	1.43	0.32
Ankle	8.79±2.74	6.5±1.95	-2.29 [1.59, 2.99]	−26.01%	7.14±2.39	5.71±2.08	-1.43 [1.37, 1.49]	20.00%	1.86	0.45
Knee	12.00±1.23	8.07±3.44	-5.74 [-13.33, 1.62]	−32.75%	11.21±5.29	7.86±3.88	-3.85 [2.73, 4.97]	-29.87%	4.79	021

Notes: Data were mean±standard deviation.

Δ: Post-training minus Pre-training. Δ (%): Δ/Pre-training. ES: Effect size (Cohen’s d). TE: Traditional exercise. VRE: Virtual reality exercise.

## Discussion

Our RCT demonstrated that VRE had a significant positive impact on attention, as indicated by higher scores for increasing patient vigor than TE did. Furthermore, VRE yielded slightly higher scores in increasing participants’ vigor than TE did. In terms of decision-making, a significant main effect of exercise type was observed, with VRE leading to higher scores than TE. However, it is worth noting that TE resulted in higher memory scores than VRE.

Our results showed VRE immediately following PA might have a more favorable impact on attention than TE. This finding is in line with prior research demonstrating the ability of VR to enhance cognitive function, particularly attention, and cognitive training to improve daily activities. ^40^Additionally, this aligns with Neumann *et al*. ^41^ who discovered that VR may not always be the optimal approach for diverting attention from exercise-related cues, thus emphasizing the importance of attentional mechanisms in shaping emotional and motivational responses to exercise in a VR environment. By directing attention towards the virtual environment, VR systems can increase the likelihood of positive impact and satisfaction, underscoring the crucial role of attentional mechanisms in shaping emotional and motivational responses during VRE. According to a 2020 research, VR environments can be customized to simulate real-life situations that require specific cognitive abilities, such as memory, decision-making, and problem-solving. ^40^ The present study investigated the acute effects of VRE on memory and compared them with those of TE alone. The current findings revealed a significant difference in memory scores between pre- and post-training, suggesting that time had a considerable impact on participants’ memory. Interestingly, memory scores varied significantly between the TE and VRE conditions, with participants’ mean response memory being higher for TE than for VRE. However, the interaction between exercise type and time was not significant, indicating that the impact of exercise type on memory did not depend on whether the memory was measured before or after the exercise. The results differ from previous research on memory in elderly stroke survivors, which suggests that training environments can be customized to improve cognitive abilities, including memory, that influence daily activities. ^40^Additionally, the results highlight the potential benefits of immersive VR in facilitating the transfer of learned abilities from the virtual world to the real world. While some studies suggest that VRE may not be as effective as TE in enhancing memory, others have identified that VRE can be helpful in reducing memory decline in older adults. ^42^ Further research is necessary to confirm these findings and identify the potential advantages of exercise for memory enhancement. Therefore, the potential benefits of VRE in memory enhancement warrant further investigation.

Evidence suggests that there is a link between attention, memory, and vigor immediately after exercise. We reported a significant main effect of exercise type on vigor, with participants reporting higher levels of vigor after VRE than after TE. Moreover, there was a significant main effect of time on participants’ vigor, with higher levels of vigor reported after exercise than before. However, there was no significant interaction between exercise type and time on participants’ vigor. Our findings are consistent with previous research indicating that acute exercise can enhance feelings of vigor in the short term. ^13^However, our results did not align with the findings of Adhyaru *et al*., ^43^ who reported no significant difference in vigor between VRE and TE.

The present study identified a significant main effect of exercise type on participants’ decision-making, with higher scores observed in VRE than TE, suggesting that VRE may enhance decision-making abilities. However, no significant main effect of time on decision-making scores was found, indicating that the effect of exercise on decision-making did not differ significantly between pre- and post- exercise measures. Our finding diverges from previous studies that reported improvements in decision-making following acute exercise, such as Ulas *et al*. ^44^ who found a single bout of moderate-intensity exercise improved decision-making performance in healthy adults. Our results are consistent with those of Shaw *et al*., ^45^ who reported that the use of a competitive virtual trainer while riding at a moderate-vigorous intensity did not improve cognitive performance. Conversely, our findings align with those of Ertoy Karagol *et al*. ^46^ who reported that VRE led to a significant improvement in vigor compared to the control group, as well as a significant increase in PA compared to the control group. These results suggest that VRE may be an effective intervention for improving vigor and PA in adolescents. Furthermore, the interaction effect between exercise type and time indicated that the style of exercise and the measurement time had different effects on decision-making abilities. Thus, further investigation, such as post-hoc tests, is necessary to identify conditions that vary significantly from one another.

This study identified significant differences in decision-making errors based on exercise type and time, indicating both have an impact. Participants made more errors during TE than during VRE, suggesting that VR provides greater cognitive benefits for decision-making. Moreover, participants made fewer errors post-training, indicating that exercise reduced decision-making errors overall. The significant interaction between exercise type and time demonstrated the effect of exercise on errors differed pre- and post-exercise. The benefits of VRE on errors seemed more pronounced post-training, while benefits of TE were less evident. Overall, these findings suggest that VRE may be more beneficial than TE for improving decision-making accuracy and that the effects of exercise on decision-making may vary depending on the type of exercise and timing of assessment. Our findings that VRE may be more advantageous than normal exercise for enhancing decision-making accuracy is supported previous study. ^47^ Mestre *et al*. ^47^ compared indoor cycling exercise with VR feedback vs. non-VR feedback and highlighted that cycling exercise with VR feedback had a reduced perceived exertion and an increased level of enjoyment of PA. This finding unequivocally demonstrates the beneficial effects of VR feedback and a virtual coach on the enjoyment of PA, with the virtual coach showing the greatest gains. ^47^

Our results indicate that VRE with bike has the potential to acutely reduce muscle spasticity. VRE with bike provide visual and auditory stimulation in addition to cycling, which may distract users and draw attention away from muscle sensations. ^17^ This reduction in sensory input could decrease muscle spasms and contractions, resulting in temporary relief from spasticity. ^43^ The immediate effects observed after using the VRE bike ^17^ suggested an acute impact on spasticity. However, longer-term investigations are needed to determine whether regular use of VRE with bike can yield sustained benefits for reducing spasticity. ^17^

These results emphasize the potential advantages of incorporating VRE as a promising intervention, with larger effect sizes, to enhance the physical well-being and motor skills of individuals with CP.

### Limitations

Somelimitations must be considered. The major one was the retrospective registration of our RCT. Since the primary purpose of trial registration is to enhance transparency, reduce bias, and promote the integrity of clinical research, retrospective registration of an RCT can pose some challenges and may be viewed with skepticism by journal editors, peer reviewers, and readers. Retrospective registration of an RCT can raise concerns about selective reporting, outcome switching, and potential bias. However, we have registered our RCT in the aim to be transparent about the timeline of the study. Registration was performed immediately as possible just before the trial ended. We included the full study protocol with detailed information on the study design, primary and secondary outcomes, and statistical analysis plan. This demonstrate that our study was conducted in a rigorous and pre-planned manner. We confirm that the present study adhered to ethical guidelines and that the retrospective registration does not compromise the ethical conduct of the trial. We explicitly discuss how we have mitigated the risk of bias in our study by addressing concerns related to selective outcome reporting and data-driven decisions. We attest that there were no modifications to the study design or analysis plan after the data collection. Second, the study’s short duration precludes conclusions about prolonged effects. Third, the “small” but calculated sample size may limit generalizability of the results. Finally, potential confounding factors such as participant exercise history and spasticity severity were not considered. Despite these limitations, the present study has enhanced our understanding of the relationship between TEs and VREs in adolescents with CP. We believe that our findings will stimulate further investigations in this important area.

### Implications and futures directions

For adolescents with CP, VRE bike games using VirZoom’s VZfit appear to be an effective alternative to boost adherence and, consequently, enhance mood. Future studies should be conducted in this regard to investigate additional topics related to VRE bike games for children, including assessing those with CP using objective measures to determine how long the virtual headset workouts last, assessing the interactions between individual and group activities, and determining the long-term effects of the activities performed both in the lab and at home.

## Conclusions

VRE can help improve attention, vigor, and decision-making in college students with CP more than TE. However, TE may be more beneficial in enhancing memory scores. Future research with larger samples sizes is needed to validate our findings and identify the mechanisms driving differences in cognitive outcomes between VR and TE. Overall, both types of exercise showed potential for improving mental performance and well-being.

## Data availability

### Underlying data

Zenodo: Data of 14 participants in the RCT titled Acute effects of virtual reality exercise bike games on psycho-physiological outcomes in college North-African adolescents with cerebral palsy [Dataset]. Zenodo. https://doi.org/10.5281/zenodo.13952330. ^48^

The project contained an Excel file that included the numerical data of the 14 participants.

### Reporting guidelines

CONSORT checklist for: Acute effects of virtual reality exercise bike games on psycho-physiological outcomes in college North-African adolescents with cerebral palsy: A randomized clinical trial. Zenodo. https://doi.org/10.5281/zenodo.10157607. ^49^

Data are available under the term Creative Commons Zero (CC0).
