# Supplementary material for: Acute effects of virtual reality exercise bike games on psychophysiological outcomes in college North-African adolescents with cerebral palsy: A randomized clinical trial
Source: F1000Res. 2025 Jul 18;12:1597. Originally published 2023 Dec 15. [Version 3] doi: 10.12688/f1000research.143189.3 (PMC11745216; doi:10.12688/f1000research.143189.3)
Supplement: Peer review history

# Reviewer report: Reviewer response for version 3

**DOI:** 10.5256/f1000research.183944.r399210

**Published:** 2025-08-11

**Authors:** Qamar Mahmood

**Affiliations:** 1 Ex-HOD, Physical Therapy Department, National Institute of Rehabilitation Medicine, Islamabad, Pakistan; 2 Director, Physiotherapy Department, Institute of Medical Rehabilitation, Islamabad, Pakistan; 3 Professor of Physical Therapy, FRAHS, Riphah International University, Islamabad, Pakistan

Author has made useful changes in revision, so no further changes are needed from my side. Article can be accepted for indexing in current state.

Is the work clearly and accurately presented and does it cite the current literature?

Yes

If applicable, is the statistical analysis and its interpretation appropriate?

I cannot comment. A qualified statistician is required.

Are all the source data underlying the results available to ensure full reproducibility?

Yes

Is the study design appropriate and is the work technically sound?

Yes

Are the conclusions drawn adequately supported by the results?

Yes

Are sufficient details of methods and analysis provided to allow replication by others?

Yes

Reviewer Expertise:

Pediatric rehabilitation specifically in the management of Cerebral Palsy, Physical therapy

I confirm that I have read this submission and believe that I have an appropriate level of expertise to confirm that it is of an acceptable scientific standard.

# Reviewer report: Reviewer response for version 2

**DOI:** 10.5256/f1000research.173736.r349413

**Published:** 2025-01-17

**Authors:** Taha Yassine Temlali

**Affiliations:** 1 Radboud University, Nijmegen, The Netherlands

The article examines the effects of two cycling exercise methods—traditional cycling using a stationary bike with a TV-based virtual cycling video and immersive virtual reality exercise (VRE) using a VR headset—on acute cognitive outcomes, vigor, and muscle spasticity in adolescents with cerebral palsy (CP). The study contributes to the field by exploring the potential of VRE to enhance cognitive functions such as attention, vigor, and decision-making, with promising results suggesting VRE may outperform traditional cycling in these areas. The authors also provide valuable insights into how virtual environments can support physical activity in this population. However, several areas could be strengthened.

 Your manuscript would benefit from referencing existing literature to support the statements made throughout the paper. Several assertions are presented without citations, which weakens the overall credibility of your arguments. By integrating appropriate references, you would not only strengthen your claims but also clearly position your findings within the broader context of existing research, offering a stronger foundation for your conclusions. Additionally, it is important to report both significant and non-significant results accurately, as non-significant findings also contribute to the understanding of the topic.

 The message and argumentation in the paper could be made more cohesive. At the moment, some of the points seem disconnected and not fully developed. A more structured approach would help clarify the overall message and enhance the impact of your conclusions. A more focused argumentation would allow each point to contribute meaningfully, resulting in a stronger, well-supported narrative throughout the manuscript.

 In its current form, I believe the article can only be considered for indexing after major revisions, particularly in terms of strengthening the argumentation and ensuring clarity and proper citation of relevant research

**Please see the comments below:**

**Introduction**

 First, while VR is introduced as a promising tool for rehabilitation, the rationale for its application in CP rehabilitation could be further developed. Including details about how VR has been previously used in similar populations, such as individuals with other neurodevelopmental disorders, and identifying gaps in current use would provide a more robust foundation for the study. Specific examples of VR interventions in CP or related populations could also help underscore its potential impact on physical activity and rehabilitation. Additionally, expanding on why VR might be more effective or appealing than traditional exercise for individuals with CP would strengthen the argument.

 Clarification of the population under study is another area for improvement. The introduction mentions university students with CP but does not explain why this group is the focus. Is the choice motivated by unique challenges faced by this demographic, or is it due to a lack of research in this age group? This should be explicitly stated. Additionally, the term "university students" might imply young adults rather than adolescents. If the study targets a specific developmental stage, such as adolescents, this should be clarified to ensure relevance and accuracy in describing the population.

 The study’s novelty and relevance could be better emphasized. While the introduction mentions investigating acute effects of VR exercise, it does not explain why this focus is particularly important. Providing more context on the differences between acute and chronic exercise effects in CP would help justify this approach. Moreover, linking the study's findings to potential advancements in rehabilitation practices or future interventions would highlight its significance. For instance, how could this study build on or inform clinical practices or the development of VR technologies tailored to the needs of individuals with CP? Incorporating references to support claims about the innovative integration of VR in CP rehabilitation would further substantiate this argument.

 Key terms like Virtual Reality Exercise (VRE) and Traditional Exercise (TE) require clearer definitions. the study appears to inaccurately categorize one condition as "traditional cycling," despite incorporating a virtual component through a TV-based cycling video. This setup represents a basic virtual environment rather than a traditional exercise. Revising the terminology to accurately reflect the study design is crucial, and providing evidence from the literature to support the chosen terminology would strengthen the manuscript's credibility.

 The claim about the novelty of implementing the VirZoom VRE system  and studying acute effects could be enhanced by citing reviews or meta-analyses that emphasize the lack of VR-based interventions for adolescents with CP. Providing evidence that highlights this gap in the literature will make the argument more compelling and underscore the significance of the study.

 By addressing these areas, the introduction could provide a more comprehensive and compelling foundation for the study, ensuring clarity, proper contextualization, and stronger alignment with the study’s aims.

**Methods**

 The methods section provides a detailed overview of the study design and procedures, but several areas lack references to substantiate key claims. Addressing these gaps with appropriate citations will enhance the credibility and robustness of the section.

 Please ensure that all variables assessed in this study are mentioned, along with the specific tests used for each of them.

 Game Selection and Rationale:

 The rationale for selecting specific games, which focus on immersive, interactive, and engaging experiences, needs to be backed by literature. Citing studies that explore the impact of such games on exercise adherence and participation in similar populations, particularly individuals with CP or other neurological conditions, would strengthen the argument for these choices. Furthermore, including references to guidelines or frameworks that highlight the criteria for selecting effective games (e.g., targeting specific muscle groups or promoting cognitive and social benefits) would validate the approach.

 Examples of lack of References for Key Claims:

 The text mentions the use of the VirZoom VRE system for cognitive stimulation, social interaction, and health outcomes but does not provide citations. Including studies or reviews that discuss these benefits in VR-based rehabilitation would establish credibility.

 General statements about the entertainment value of VR influencing exercise adherence and participation should be supported by research. Adding references to studies that demonstrate how entertainment and engagement impact adherence would make these assertions more persuasive.

 Customization and Rehabilitation Objectives:

 The statement regarding tailoring exercise experiences to individual rehabilitation objectives is an important point but would benefit from references to studies or examples that illustrate how customization in VR-based systems has been effective in rehabilitation contexts. Such evidence would demonstrate that the study’s approach aligns with best practices in the field.

**Analysis**

 Assumption Testing

 No mention is made of other standard assumptions for ANOVA, such as normality and homogeneity of variances.

**Results**

 Attention

 The reporting of results for attention lacks clarity and precision. The interaction effect is described as statistically significant (F(1,13)=6.30, p=0.026, ηp²=0.327), but these same values are also attributed to the main effect of exercise type, creating confusion about which effect is being discussed. Participants’ attention scores are stated to be higher in VRE compared to TE, but the actual means are missing, with only standard deviations provided, hindering a complete interpretation.

 Grammatical inconsistencies, such as the awkward phrasing "Participants’ mean response attention was higher," further obscure the findings and could be revised for clarity. The main effect of time (F(1,13)=97.066, p=0.001, ηp²=0.882) is reported as significant, but mean scores for pre- and post-training are absent. Additionally, the practical significance and implications of the findings, particularly the relationship between exercise type and attention, are not adequately explained, leaving their relevance unclear.

**Memory**

 Several issues detract from the clarity of the memory results. The main effect of exercise type (F(1,13) = 3.059, p = 0.104, ηp² = 0.190) and the interaction effect (F(1,13) = 3.59, p = 0.104, ηp² = 0.190) are incorrectly described as statistically significant, despite a p-value of 0.104 not meeting the conventional significance threshold (p < 0.05). Misinterpreting p-values in this manner can mislead readers.

 The absence of mean scores for memory in the TE and VRE conditions further hinders the interpretation, as the claim that memory was higher in TE compared to VRE is unsupported by sufficient data; only standard deviations (SDs) are reported. The main effect of time (F(1,13) = 180.974, p = 0.001, ηp² = 0.933) is validly described as significant, and it is the only result meeting significance criteria.

 Grammatical errors, such as "participants’ mean response memory were higher," should be corrected to "participants’ mean response memory was higher" to maintain proper subject-verb agreement. To improve readability, results should be reorganized into distinct paragraphs for main effects and interactions, explicitly stating which findings are significant and ensuring clarity in reporting.

**Total Score**

 Several aspects of this section could be clarified to improve accuracy and readability. The interaction effect (F(1,13) = 19.118, p = 0.001, ηp² = 0.595) is identical to the main effect of exercise type, suggesting a possible reporting error. Additionally, while standard deviations (SDs) for VRE and TE conditions are provided, the corresponding means are missing, limiting the ability to make meaningful comparisons.

 There is inconsistency in terminology, as the sentence "Descriptive statistics revealed that participants’ mean response attention was higher post-training" appears to incorrectly use "attention" instead of "total score." Furthermore, phrasing such as "Participants’ mean response total score were higher" should be revised for grammatical accuracy. To strengthen this section, it is essential to provide all key data points, including means, ensure precise terminology, and avoid redundancy in statistical reporting.

**Vigor**

 The reporting of results for vigor is unclear and includes inaccuracies. Non-significant findings (F(1,13) = 2.576, p = 0.133, ηp² = 0.165) are described as meaningful, which is not true. Additionally, mean values are absent, and the provided standard deviations (SDs) are insufficient for meaningful comparisons. The term "statistically significant interaction" is incorrectly applied to a non-significant result (p = 0.133), further contributing to the inaccuracy.

**Decision-Making Correct**

 While the significant main effect of exercise type (F(1,13) = 26.338, p = 0.01, ηp² = 0.670) is noted, the absence of mean values alongside SDs limits interpretability. The main effect of time (F(1,13) = 3.833, p = 0.72) is not significant but is misinterpreted as meaningful. Additionally, there is a labeling error where "mean response attention" is used instead of "decision-making," which needs correction. Consistent reporting of statistical values and terminology would clarify the findings.

**Decision-Making Errors**

 Significant results (F(1,13) = 28.202, p = 0.001, ηp² = 0.684) are presented without accompanying means, making SD comparisons incomplete. While the main effect of time (F(1,13) = 5.350, p = 0.038, ηp² = 0.292) is accurately reported, missing data (e.g., pre- and post-training means) undermines clarity.

**MAS**

 This section highlights reductions in spasticity for TE and VRE groups, but vague language like "more substantial improvements" detracts from objectivity. The lack of statistical results to support these claims leaves readers uncertain about their validity. Including consistent numerical data (means, SDs, and test statistics) would enhance the reliability and precision of this section.

**Overall, these sections would benefit from clear and consistent reporting of means, SDs, and test statistics, precise terminology, and a focus on significant findings to improve the clarity and accuracy of the reported results.**

 Discussion

**Inaccurate or Unsubstantiated Statements**

 The paragraph lacks clear connection between the findings from Neumann et al. and the results of your study. Specifically, the reference to Neumann et al. seems somewhat disconnected, as their conclusion about VR not always being optimal for diverting attention from exercise-related cues does not directly align with your claim that VRE improves attention. The phrasing also suggests that VR increases the likelihood of positive impact and satisfaction, but this claim could benefit from more specific evidence or examples to support it. Additionally, the paragraph could be more concise by avoiding repetition, particularly in the mention of "attentional mechanisms" which is emphasized multiple times without adding new information. The relationship between the attention measured in your study and the statement "By directing attention towards the virtual environment, VR systems can increase..." is unclear. It would be helpful to clarify how the attention outcomes observed in your study specifically link to the direction of attention in the virtual environment and how this contributes to increased attention and satisfaction. Lastly, the connection between VR and attentional mechanisms is somewhat vague—clarifying how VR specifically impacts attention in your study would strengthen the argument.

 Some statements in the text lack alignment with the study's scope or findings, leading to inaccuracies. For example, the claim that “Our findings that VRE may be more advantageous than normal exercise for **enhancing decision-making accuracy** is supported previous study. ^47^ Mestre *et al*. ^47^ compared indoor cycling exercise with VR feedback vs. non-VR feedback and highlighted that cycling exercise with VR feedback had a reduced **perceived exertion and an increased level of enjoyment** of PA” is not accurate. The referenced study (Mestre et al.) explores perceived exertion and enjoyment and not decision making accuracy, so how does that study support your finding of enhanced decision making accuracy? Such strong conclusions should be either removed or referenced properly with studies that actually support your exact findings.

 Similarly, the statement, “VRE immediately following PA might have a more favorable impact on attention than TE,” implies an effect that is not explicitly tested or supported by the methodology. To ensure accuracy, it would be better to clearly describe the timing of measurements and avoid speculative claims.

 Another example is the assertion, “These results emphasize the potential advantages of incorporating VRE as a promising intervention... to enhance the physical well-being and motor skills of individuals with CP.” Since the study focuses on cognitive outcomes rather than motor skills or physical well-being, this statement should be removed or revised to avoid unsupported claims.

 The suggestion that “VRE with bike provides visual and auditory stimulation... which may distract users and draw attention away from muscle sensations” is plausible but not substantiated by the study’s results. To maintain credibility, the discussion should focus on observed outcomes and refrain from over-interpreting mechanisms without evidence or reference studies supporting your arguments.

**Over-generalizations**

 Several statements in the text overreach beyond the study’s findings. For instance, “Evidence suggests that there is a link between attention, memory, and vigor immediately after exercise” is not supported by references and is overly broad and not directly addressed in the study. It would be more appropriate to focus on the relationships explicitly tested and observed or backed up by evidence from literature .

**Questionable Interpretations of Results**

 example:Some interpretations of the study’s results are inconsistent or unclear. For example, the memory scores are described in conflicting terms, with significant differences mentioned in one part and a lack of significant interaction between exercise type and time stated in another. These contradictions should be clarified by revisiting the statistical results and ensuring consistency. And by giving appropriate explanation for this.

**Defensive Tone in Limitations**

 The section on retrospective registration comes across as overly defensive, which detracts from the objectivity of the discussion. Instead of justifying this limitation extensively, it would be more effective to briefly acknowledge the retrospective registration and highlight measures taken to mitigate potential bias. This approach ensures transparency without appearing overly apologetic.

**Lack of Alignment Between Findings and Conclusions**

 Certain recommendations for future research do not align with the study’s scope. For instance, the suggestion to investigate the long-term effects of the activities is valid but not directly supported by the current study, which focuses on short-term outcomes. To maintain alignment, this recommendation should be framed as exploratory and emphasize directions based on the study’s findings.

Is the work clearly and accurately presented and does it cite the current literature?

Partly

If applicable, is the statistical analysis and its interpretation appropriate?

Partly

Are all the source data underlying the results available to ensure full reproducibility?

Yes

Is the study design appropriate and is the work technically sound?

Yes

Are the conclusions drawn adequately supported by the results?

Partly

Are sufficient details of methods and analysis provided to allow replication by others?

Yes

Reviewer Expertise:

My expertise lies in exercise physiology, exergames, and physical activity research. While I have a strong foundation in these areas, my experience with statistics is limited, and I do not specialize in statistical analysis

I confirm that I have read this submission and believe that I have an appropriate level of expertise to confirm that it is of an acceptable scientific standard, however I have significant reservations, as outlined above.

# Reviewer report: Reviewer response for version 2

**DOI:** 10.5256/f1000research.173736.r343915

**Published:** 2024-11-28

**Authors:** Qamar Mahmood

**Affiliations:** 1 Ex-HOD, Physical Therapy Department, National Institute of Rehabilitation Medicine, Islamabad, Pakistan; 2 Director, Physiotherapy Department, Institute of Medical Rehabilitation, Islamabad, Pakistan; 3 Professor of Physical Therapy, FRAHS, Riphah International University, Islamabad, Pakistan

I am well and fully satisfied with all the changes made by author in response to comments. I appreciate author 'efforts in this regard. So from my side, no further changes are required.

 This manuscript is ready to be indexed now.

Is the work clearly and accurately presented and does it cite the current literature?

Yes

If applicable, is the statistical analysis and its interpretation appropriate?

I cannot comment. A qualified statistician is required.

Are all the source data underlying the results available to ensure full reproducibility?

Yes

Is the study design appropriate and is the work technically sound?

Yes

Are the conclusions drawn adequately supported by the results?

Yes

Are sufficient details of methods and analysis provided to allow replication by others?

Yes

Reviewer Expertise:

Pediatric rehabilitation specifically in the management of Cerebral Palsy, Physical therapy

I confirm that I have read this submission and believe that I have an appropriate level of expertise to confirm that it is of an acceptable scientific standard.

# Reviewer report: Reviewer response for version 1

**DOI:** 10.5256/f1000research.156823.r237583

**Published:** 2024-05-30

**Authors:** Hsiu-Ching Chiu

**Affiliations:** 1 I-Shou University, Kaohsiung, Taiwan

**General comments**

 The topic under investigation is interesting, but there are some fundamental problems, such as the design of intervention. Furthermore, authors should improve the format of the presentation, suggesting having an example of published RCT paper to follow.

**Abstract.**

 Not clear because of no information about the intervention such as weeks.

**Introduction-content**

 Not read because method is very confusing.

**Methods:** **general**

 -Ethics considerations have been already mentioned in the design, so it would be unnecessary to repeat.

 -Randomization should be part of design, not separate paragraph.

**Methods:** **design**

 Even this study has been registered, authors still need to provide information about design, such as training and outcomes etc.

**Methods:** **Figure 1**

 Authors should show how many weeks of intervention as well as a brief content for both groups.

**Methods:** **participants**

 -should use “Participants” to replace “Patients”.

 -Actual number of participants and characteristics should be in the results.

 Methods: Intervention

 This is main problem because it’s hard to believe it will be effective for only 2 times training.

**Results:** **Table 1 (missing). Characteristic of participants**

**Results:** **Table 2 (missing).Differences between groups in all Outcomes across times**

**Discussion and conclusion:**

 Not read because method and results are very unstructured.

**Readability and style.**

 This paper is not easy to read, because of unstructured and the fluency of English. For example, “….in adolescents with CP in North African college….” Not “In North African college adolescents with CP”. Another example, it may be clearer as “The Experimental group undertook xx …times a week for xx week….” Not “ Patients underwent two…” in the method.

Is the work clearly and accurately presented and does it cite the current literature?

No

If applicable, is the statistical analysis and its interpretation appropriate?

I cannot comment. A qualified statistician is required.

Are all the source data underlying the results available to ensure full reproducibility?

No

Is the study design appropriate and is the work technically sound?

No

Are the conclusions drawn adequately supported by the results?

No

Are sufficient details of methods and analysis provided to allow replication by others?

No

Reviewer Expertise:

My past research program was in understanding the relative contribution of motor impairments to activity limitation and participation in people with hemiplegic cerebral palsy (CP). I have finished one Cochrane review entitled as “Mechanically assisted walking training for walking, participation, and quality of life in children with cerebral palsy” for children with CP by examining outcomes across all domains of the International Classification of Functioning, Disability and Health (ICF).

I confirm that I have read this submission and believe that I have an appropriate level of expertise to confirm that it is of an acceptable scientific standard, however I have significant reservations, as outlined above.

# Response

**Published:** 2024-10-19

**Authors:** Amine Ghram

**Affiliations:** Cardiac rehabilitation, Hamad Medical Corporation, Doha, Doha, Qatar

**Reviewer Comment 1:** *The topic under investigation is interesting, but there are some fundamental problems, such as the design of intervention. Furthermore, authors should improve the format of the presentation, suggesting having an example of a published RCT paper to follow.*

**Response:**

 We appreciate your feedback regarding our manuscript. We recognize the necessity for a more precise intervention design and consulted well-structured randomized controlled trial (RCT) papers to enhance the format and presentation of our work. Furthermore, we meticulously revised the intervention description to ensure adherence to the standard RCT formats.

**Action Taken:**

 The intervention section has been revised to adhere to a more structured format commonly used in published randomized controlled trials (RCTs). This revision encompasses a clear delineation of the study design, intervention details, and outcome measures to enhance the methodological clarity and rigor.

**Reviewer Comment 2:** *Abstract not clear because of no information about the intervention such as weeks.*

**Response:**

 We acknowledge your observation and have subsequently revised the abstract to incorporate specific details regarding intervention duration, thereby ensuring comprehensive information about the study's structure for readers.

**Action Taken:**

 The abstract now incorporates the following information (Lines 30–32): " **The intervention consisted of a single 40-minute session of virtual reality exercise (VRE) compared to traditional exercise (TE), conducted in a controlled laboratory environment** **.**" This addition provided essential details regarding the intervention and enhanced the comprehensiveness of the abstract.

**Reviewer Comment 3:** *Introduction not read because method is very confusing.*

**Response:**

 We acknowledge the ambiguity engendered by the initial organization of the methods section. To rectify this issue, we have restructured both the Introduction and Methods sections to enhance the clarity and logical progression.

**Action Taken:**

 The Introduction has been revised to ensure enhanced alignment with the study's methodology and objectives. Furthermore, the Methods section provides a comprehensive, sequential explanation of the intervention and study design, thereby minimizing potential ambiguities.

**Reviewer Comment 4:** *Method: - Ethics considerations have been already mentioned in the design, so it would be unnecessary to repeat. - Randomization should be part of design, not a separate paragraph.*

**Response:**

 We would like to acknowledge this recommendation. Redundant ethical considerations were eliminated, and the randomization process was integrated into the study design section to enhance the coherence of the presentation.

**Action Taken:**

 The randomization protocol has been relocated to the design section (lines 175–176), and superfluous ethical considerations have been eliminated to enhance conciseness and clarity.

**Reviewer Comment 5:** *Method: Even this study has been registered, authors still need to provide information about design, such as training and outcomes, etc.*

**Response:**

 We acknowledge your observation and have provided supplementary information concerning the study design, encompassing training protocols and specific outcomes.

**Action Taken:**

 The Methods section now delineates the training regimen, outcome measures, and data collection processes, thereby ensuring that the study design is fully transparent and adheres to RCT guidelines.

**Reviewer Comment 6:** *Methods: Figure 1 - Authors should show how many weeks of intervention as well as a brief content for both groups.*

**Response:**

 Thank you for your suggestion. The intervention duration was clarified, and a concise description of the intervention for both groups was provided (Figure 1).

**Action Taken:**

 Figure 1 demonstrates that the intervention was implemented as a single-session trial and a concise description of the intervention protocols for both groups was provided.

**Reviewer Comment 7:** *Methods: participants - Should use “Participants” to replace “Patients”. - Actual number of participants and characteristics should be in the results.*

**Response:**

 We agree with the reviewer's recommendations. The term "participants" has been substituted for "patients" throughout the manuscript, and the precise number of participants, along with their demographic characteristics, has been moved to the Results section.

**Action Taken:**

 The term "patients" has been replaced with "participants" throughout the manuscript, and the characteristics of participants have been incorporated into the results section (Lines 301, Table 1).

**Reviewer Comment 8:** *Methods: Intervention - This is the main problem because it’s hard to believe it will be effective for only two times training.*

**Response:**

 This study has several limitations. While the present investigation focused on the immediate effects of a single session, further studies are necessary to assess the long-term impact of repeated interventions. This limitation has been addressed in the Discussion section.

**Action Taken:**

 This study acknowledges this limitation and suggests that future research should incorporate extended interventions to evaluate the cumulative effects of repeated VRE sessions.

**Reviewer Comment 9:** *Results: Table 1 (missing). Characteristics of participants.*

**Response: **We acknowledge your observation and have subsequently incorporated Table 1, which delineates the participants' characteristics including age, sex, and pertinent demographic data.

**Action Taken: **Table 1 has been incorporated into the Results section, presenting the characteristics of the study participants as requested.

**Reviewer Comment 10:** *Results: Table 2 (missing). Differences between groups in all outcomes across times.*

**Response: **Table 2 presents the differences between the groups across all outcome measures and time points.

**Action Taken: **Table 2 has been added to the Results section to show the differences in outcomes between the traditional exercise and VRE groups.

**Reviewer Comment 11:** *The discussion and conclusion have not been read because the method and results are unstructured.*

**Response: **The Methods and Results sections have been restructured to enhance the clarity and logical progression. The discussion has been aligned to provide a coherent narrative that establishes connections among the methods, results, and conclusions.

**Action Taken: **The Methods, Results, and Discussion sections were restructured to enhance clarity, thereby improving the accessibility and logical organization of the manuscript.

**Reviewer Comment 12:** *This paper is not easy to read because of unstructured format and fluency of English.*

**Response: **The authors acknowledge concerns regarding readability and fluency. The manuscript has undergone a comprehensive revision to enhance both the structure and fluency of the English language, thereby ensuring a more lucid and professional presentation.

**Action Taken: **The manuscript has undergone comprehensive revision to enhance clarity, grammatical accuracy, and overall fluency, thereby improving readability while maintaining scholarly rigor.

 Thank you for your valuable feedback. We believe that these revisions have significantly improved the clarity, structure, and scientific rigor of our manuscript. We look forward to your feedback, and hope that the revised manuscript meets your expectations.

# Reviewer report: Reviewer response for version 1

**DOI:** 10.5256/f1000research.156823.r248808

**Published:** 2024-03-29

**Authors:** Qamar Mahmood

**Affiliations:** 1 Ex-HOD, Physical Therapy Department, National Institute of Rehabilitation Medicine, Islamabad, Pakistan; 2 Director, Physiotherapy Department, Institute of Medical Rehabilitation, Islamabad, Pakistan; 3 Professor of Physical Therapy, FRAHS, Riphah International University, Islamabad, Pakistan

This article investigated the immediate effects of virtual reality exercise (VRE) bike games on young adults in North Africa with cerebral palsy (CP).

 Its brief description is as under:

- **Study Design:** Randomized controlled trial (considered a gold standard for clinical research)
- **Participants:** 14 college students (average age 15.6) diagnosed with CP
- **Intervention:** Participants were divided into two groups. One group exercised using VR bike games for 40 minutes (VRE group), while the other group participated in traditional exercise (TE group) for the same duration.
- **Outcomes:** The study measured changes in attention, vigor (energy levels), and decision-making abilities after the exercise sessions.
- **Key Findings:** Compared to traditional exercise, VR exercise led to significant improvements in attention and vigor.

**Its Clinical Relevance:**

 Cerebral palsy can impair both physical function and mental well-being. This study suggests VR exercise could be a valuable tool for improving cognitive function and motivation in young adults with CP. VR's engaging nature might make exercise more enjoyable and help individuals with CP adhere to exercise routines.

 However, it's important to note that the study only looked at short-term effects. More research is needed to confirm these findings and explore the long-term benefits of VR exercise for CP management.

 This article is well written, however following major and minor changes will improve its quality for publication.

**Minor Changes needed:**

- ***In abstract section;*** Briefly mention the mixed findings on memory to provide a more balanced picture of the results.

- ***In introduction section:*** **Emphasize the *novelty and significance* of the present study. Instead of stating existing knowledge about the benefits of regular exercise, briefly mention the *limited research on the effects of acute exercise on cognitive function in adolescents with CP*, highlighting the gap your study aims to address.
- Consider reorganizing the information flow. Start by highlighting the *gap in knowledge * about acute exercise and cognitive function in adolescents with CP. Then, briefly mention the existing evidence of VR's effectiveness in promoting physical activity, leading to the introduction of the VirZoom VRE bike and the research question.
- Briefly define *psychophysiological outcomes* for the audience unfamiliar with these terms.

- ***In methodology section: *** Further emphasize the *novelty * of using VirZoom VRE compared to traditional exercise bikes in this specific population (adolescents with CP).
- Briefly explain *the rationale* behind choosing specific games on the VirZoom VRE for the study.

**Major Changes needed:**

**

***a- **In** result section, please make following changes.***

- Demographic characteristics should be presented as group wise. Because you have two groups, therefore, split age, gender etc. group wise. You can make three groups as TE, VRE and Total study population too.
- Mention the name of statistical test which you have used in stats of Table-2.
- You have mentioned that significant changes took place in Vigor too which is not the case as per stats of the study. Correct this.
- Mention the age in months instead of years because it sounds better.
- How many readings of MAS were taken on a particular joint? Why have you not used the mean of readings for MAS which is established practice in literature?
- Table -3 lacks the significance level (p value). Where is it? In absence of statistical significance, how can you claim that changes are significant?

***b-In discussion section; please make following    changes.***

- Don’t use the words our, I, my in research writings. Instead of our study, you can say present study or current study. Similarly instead of we found, you can write, results exhibits etc.
- While acknowledging limitations, the discussion could be strengthened by using more cautious language when describing the potential benefits of VRE, especially in areas where the findings are mixed or inconclusive.
- If possible, the discussion could be strengthened by delving deeper into the possible mechanisms by which VRE might be exerting its effects on various outcomes.

Is the work clearly and accurately presented and does it cite the current literature?

Yes

If applicable, is the statistical analysis and its interpretation appropriate?

I cannot comment. A qualified statistician is required.

Are all the source data underlying the results available to ensure full reproducibility?

Yes

Is the study design appropriate and is the work technically sound?

Yes

Are the conclusions drawn adequately supported by the results?

Yes

Are sufficient details of methods and analysis provided to allow replication by others?

Yes

Reviewer Expertise:

Pediatric rehabilitation specifically in the management of Cerebral Palsy, Physical therapy

I confirm that I have read this submission and believe that I have an appropriate level of expertise to confirm that it is of an acceptable scientific standard, however I have significant reservations, as outlined above.

# Response

**Published:** 2024-10-19

**Authors:** Amine Ghram

**Affiliations:** Cardiac rehabilitation, Hamad Medical Corporation, Doha, Doha, Qatar

**Reviewer Comment 1:** *In the Abstract section, briefly mention the mixed findings on memory to provide a more balanced picture of the results.*

**Response:**

 We appreciate your insightful comments. Presenting a more balanced view of results, particularly regarding memory, is crucial for accuracy and transparency. The abstract has been revised to reflect heterogeneous findings on memory, ensuring that it aligns with the overall outcomes of the study.

**Action Taken:**

 The following statement was incorporated into the abstract (lines 33–37):’ **Fourteen participants (42.9% female) were included in this analysis, and the results indicated that VRE had a statistically significant positive effect on attention and vigor compared to TE. While participants in the VRE group exhibited enhanced attention levels and reported elevated levels of vigor subsequent to the exercise sessions, the memory results did not reach statistical significance** **.**" This modification ensures a comprehensive interpretation of the findings and prepares readers for the study's conclusions.

**Reviewer Comment 2:** *In the introduction section, emphasize the novelty and significance of the present study. Instead of stating existing knowledge about the benefits of regular exercise, briefly mention the limited research on the effects of acute exercise on cognitive function in adolescents with CP, highlighting the gap your study aims to address.*

**Response:**

 Thank you for your constructive feedback. To emphasize the novelty and significance of our investigation, we restructured the introduction to focus on the limited research on the effects of acute exercise on cognitive function in adolescents with cerebral palsy (CP). This modification highlights the research gap that our study addresses and underscores the unique contribution of our work to the field.

**Action Taken:**

 The following passage was incorporated into the introduction (lines 71–78):’ **This study represents a significant advancement in elucidating the relationship between acute exercise and cognitive function in adolescents with CP. Despite extensive research demonstrating the benefits of regular exercise on cognitive health, there remains a notable gap in our understanding of how a single bout of exercise affects cognitive function in this specific population. This study was motivated by a growing body of evidence suggesting that acute aerobic exercise positively influences cognitive function, mood, and memory in various populations. However, these effects remain relatively unexplored in children and adolescents, particularly those with CP. This gap underscores the need for targeted research to elucidate how acute exercise can be utilized to enhance cognitive outcomes in this vulnerable population** **.**" This addition enhances the novelty of our study and elucidates how our research addresses a gap in the literature in accordance with the reviewer's recommendation.

**Reviewer Comment 3:** *Consider reorganizing the information flow. Start by highlighting the gap in knowledge about acute exercise and cognitive function in adolescents with CP. Then briefly mention the existing evidence of VR's effectiveness in promoting physical activity, leading to the introduction of the VirZoom VRE bike and the research question.*

**Response:**

 We acknowledge your comprehensive feedback regarding the structure of the Introduction. In accordance with your recommendation, we have restructured the introduction to initially address the gap in current knowledge concerning acute exercise and cognitive function in adolescents with cerebral palsy (CP), subsequently transitioning to existing evidence on virtual reality (VR) as a modality for promoting physical activity. This revised organization enhances the clarity of the narrative and establishes a logical progression towards the introduction of the VirZoom VRE bike and the research question.

**Action Taken:**

 The introduction now commences with a discussion of the limited research on acute exercise and cognitive function in adolescents with CP, followed by a brief mention of VR's effectiveness of VR (Lines 96–103). " **This study represents a significant advancement in understanding the impact of technology, specifically virtual reality (VR), on the well-being of adolescents with cerebral palsy (CP). Although previous studies have established the benefits of regular physical activity in adults with CP, there is a paucity of data regarding the immediate effects of exercise, particularly on cognitive performance, in adolescents with CP. The objective of our randomized controlled trial (RCT) was to compare the physiological and psychological effects of using the VirZoom VR exercise cycle with traditional exercise (TE) bicycles among university students with CP in North Africa. This study aimed to examine the impact of acute exercise on cognitive function, affection, memory, and decision-making abilities. This research contributes valuable insights into a critical topic that has received limited attention in this specific population** **.**" This reorganization provided a more coherent rationale for the study and more effectively established the context for the research question.

**Reviewer Comment 4** **:** *Briefly define psychophysiological outcomes for the audience unfamiliar with these terms.*

**Response:**

 We appreciate your recommendation. It is acknowledged that the term "psychophysiological outcomes" may not be familiar to all readers; therefore, a concise definition has been incorporated to enhance comprehension for a broader audience.

**Action Taken:**

 The following definition has been incorporated (lines 53–56):’ **Psychophysiological outcomes refer to the observable effects of psychological processes on the physiological functions of the body (8). Psychophysiology is the scientific study of how cognitive processes, emotional states, and behaviors influence physiological responses, including heart rate, blood pressure, hormone levels, and brain function (9).**" This succinct definition ensures that all readers, irrespective of their expertise, comprehend this crucial term and its significance to this study.

**Reviewer Comment 5:** *In the methodology section: Further emphasize the novelty of using VirZoom VRE compared to traditional exercise bikes in this specific population (adolescents with CP).*

**Response:**

 We acknowledge your feedback and concur that it is essential to emphasize the novelty of the VirZoom VRE system, particularly its application in adolescents with CP. The utilization of immersive VR technology in exercise interventions for this population represents a relatively nascent area of research, and we have elucidated this novelty more explicitly in the methodology section.

**Action Taken:**

 We revised the methodology section to include the following statement (Lines 128–152):’ **it is essential to emphasize the novelty of implementing the VirZoom VRE system in comparison to TE bikes, particularly within the context of adolescents with CP. Although TE bikes have been utilized in numerous rehabilitation settings, the integration of VR technology represents an innovative approach to increase physical activity and improve health outcomes in this specific population. The VirZoom VRE system provides a unique and immersive fitness experience that transcends the limitations of conventional stationary cycles by offering users dynamic virtual environments to explore during exercises. This novel approach not only introduces an element of enjoyment and engagement in the training program, but also presents opportunities for cognitive stimulation and social interaction, which are particularly beneficial for adolescents with CP. Furthermore, the VirZoom VRE system enables customizable training protocols and game-based activities to address the diverse needs and abilities of individuals with CP. By incorporating games that target specific muscle groups, movement patterns, and cognitive skills, we can tailor the exercise experience to align it with the individual rehabilitation objectives and interests of our participants. By emphasizing the novelty of implementing the VirZoom VRE system in this study, we underscore its potential to transform the delivery of physical activity interventions for adolescents with CP, ultimately leading to improved health outcomes and enhanced quality of life for this population. The selection of specific games in the VirZoom VRE system for our study is based on several rationales. Primarily, we aimed to ensure that the chosen games offered a diverse range of physical activities that engaged various muscle groups and movement patterns, addressing the heterogeneous needs and abilities of adolescents with CP. Second, we prioritized games that are immersive, interactive, and engaging, as research indicates that the entertainment value of VR experiences can significantly influence exercise adherence and participation. Additionally, we considered the cognitive demands of each game, selecting those that incorporated elements of attention, memory, decision-making, and problem solving to potentially enhance cognitive outcomes in our participants (31). Lastly, we aim to incorporate games that facilitate social interaction and competition, fostering a sense of camaraderie and motivation among participants, which may further contribute to the efficacy of the intervention**." This explanation emphasizes the innovative nature of the intervention and its potential benefits, thereby elucidating the study's contribution to the field.

**Major Changes needed:**

****a-In the result section, please make following changes.

**Response to Reviewer’s Comment**

 We appreciate the reviewer’s suggestion to present demographic characteristics in a group-wise format. This change enhanced the clarity and comprehensibility of the participants’ data.

**Action Taken:** We created a new Table 1 that presents the demographic characteristics for each group (TE, VRE) as well as for the total study population. The table is formatted as follows.

**Table 1: Demographic Characteristics of Participants (refer the below link)**

https://f1000research.s3.amazonaws.com/linked/685666.Table_1_Demographic_Characteristics_of_Participants.pdf

 This table enhances the presentation of demographic data and provides a clearer comparison between the groups in accordance with the reviewer's recommendations.

**Reviewer Comment 6**

 •          Mention the name of the statistical test that you have used in stats in Table-2.

**Response:** 

 Thank you for your valuable feedback regarding the statistical analysis of our study. You are correct that we should have explicitly mentioned the statistical tests used for the results presented in Table 2. We appreciate your attention to detail and opportunity to clarify this important aspect of our methodology.

**Action Taken:**

  We have revised the manuscript to include the specific statistical tests used for the analysis presented in Table 2. The statistical method has been added to the "Statistical analysis" section of the Methods and referenced in the table caption. Text added in text: line number 283-291 in bold **"** ****A 2 × 2 repeated-measures ANOVA was conducted to examine the effects of exercise type (Traditional Exercise vs. Virtual Reality Exercise) and time (Pre vs. Post) on performance scores. Data were analyzed using SPSS version 28.0. Prior to analysis, Mauchly's test of sphericity was performed to assess the assumption of sphericity. If Mauchly's test was significant, indicating a violation of sphericity, the degrees of freedom were corrected using the Greenhouse-Geisser or Huynh-Feldt corrections, as appropriate. Main effects and interactions were evaluated, and post hoc tests were conducted using the Bonferroni correction to further explore the significant effects. Descriptive statistics, including means and standard deviations for each condition, were calculated to provide a comprehensive understanding of the data. Statistical significance was set at an alpha level of .05 for all analyses.** **)."**** Additionally, we updated the caption for Table 2 : line number 308 in **"Table 2. Comparison of pre- and post-training scores for attention, memory, total score, vigor, correct decision making, and decision-making errors in both the TE and VRE training conditions using 2 × 2 repeated-measures ANOVA."**We believe that these additions have clarified the statistical method used and addressed your concerns. The use of repeated-measures ANOVA is appropriate for our study design as it allows us to examine the effects of both exercise type and time, as well as their interaction, on our outcome measures.

**Reviewer Comment 7**

 •          You have mentioned that significant changes took place in Vigor, too, which is not the case as per the stats of the study. Correct this.

**Response:** Thank you for your valuable feedback regarding the reporting of vigor results. We appreciate your detailed attention. Upon re-evaluation of the statistical analysis, we corrected the manuscript to remove any references to significant changes in vigor.

**Action Taken:** The updated text accurately reflects that the analysis did not indicate significant changes in vigor, according to the statistical results. **The previous statement, "significant changes were observed in vigor," has been removed and replaced with "** **Exercise type had no significant changes on patient vigor** **." This correction can be found in the revised manuscript in the Results section (line 132).** We have made necessary revisions to ensure clarity and accuracy in reporting the findings. Thank you for helping us improve the quality of our manuscript.

**Reviewer Comment 8**

 •          Mention the age in months instead of years because it sounds better.

**Response:** Thank you for your suggestion regarding the reporting of participants' ages. We appreciate your attention to the details and your recommendations to enhance the precision of our demographic data.

**Action Taken:** We have revised the manuscript to report participants' ages in months instead of years. **Specifically, in the Results section, line 306, we have changed "15.6 ± 0.7 years" to "163.57 ± 41.58 months.** This modification provides a more precise representation of the participants' ages and aligns with the best practices for reporting developmental data for adolescent populations.

 We believe that this change has improved the clarity and accuracy of our demographic information. Thank you for helping us enhance the quality of our manuscript.

**Reviewer Comment 9**

 •          How many readings of MAS were taken on a particular joint? Why have you not used the mean of readings for MAS which is established practice in literature?

**Response:** Thank you for your insightful comments regarding the measurement of the Modified Ashworth Scale (MAS) readings. We appreciate your attention to methodological rigor.

**Action Taken:** In the revised manuscript, we have clarified that **three readings of the MAS were taken for each joint to ensure reliability and accuracy.** To adhere to established practices in the literature, we calculated and used the mean of these readings in our analysis. **This adjustment is reflected in the Methods section, specifically on line 278, where we now state that "The mean of three MAS readings was used for each joint assessment."** We believe that this change enhances the validity of our findings and aligns our methodology with the standard practices in the field. Thank you for your valuable feedback, which has helped us improve the quality of our manuscript.

**Reviewer Comment 10**

 •          Table -3 lacks the significance level (p value). Where is it? In absence of statistical significance, how can you claim that changes are significant?

**Response:** Thank you for your valuable feedback regarding Table 3. We appreciate your attention to the details and emphasize the importance of statistical significance in reporting the results.

**Action Taken:** We updated Table 3 to include the significance levels (p-values) for all reported outcomes. **This clarifies the statistical significance of the changes observed in the present study. Specifically, we have added p-values in the last column of Table 3, which can be found in  the revised manuscript.** Moreover, we have revised the text to ensure that any claims of significance are supported by the corresponding statistical outcomes, which enhances the transparency and rigor of our results, aligning our reporting with best practices in the literature. Thank you for your constructive suggestions, which helped us improve the quality of our manuscript.

**Reviewer Comment 11**

   b-In discussion section; please make following changes.

**Reviewer Comment 12**

 •          Don’t use the words our, I, my in research writings. Instead of our study, you can refer to the present study or the current study. Similarly instead of we found, you can write, results exhibits etc.

**Response:** We appreciate your feedback regarding the use of personal pronouns in research writing. We agree that maintaining an objective tone is important in scientific communication.

**Action Taken:** We have carefully reviewed the entire manuscript and made the following changes:

1. Replaced "our study" with "the present study" the present study’ throughout the document.
2. Changed, "we found" to "results exhibit" where applicable.
3. Replaced "we suggest" with "the current study suggests" as appropriate.
4. Removed all instances of "I" and "my" from the manuscript.

These revisions can be found throughout the document, particularly in the **Results section and Discussion section**.We believe that these changes have improved the objectivity and professionalism of our writing, while maintaining clarity. Thank you for helping us enhance the quality of our manuscript.

**Reviewer Comment 13**

 •          While acknowledging the limitations, the discussion could be strengthened by using more cautious language when describing the potential benefits of VRE, especially in areas where the findings are mixed or inconclusive.

**Response:** Thank you for your valuable feedback regarding the language used to discuss the potential benefits of Virtual Reality Exercise (VRE). We appreciate your emphasis on the importance of using cautious language, especially when addressing areas with mixed or inconclusive findings.

**Action Taken:** In response to your comment, we have thoroughly revised the discussion section to incorporate a more nuanced and careful approach in describing the potential benefits of VRE. Specifically, we have:

1. **Emphasizing the need for further research** by stating that while some findings suggest benefits, **it is crucial to recognize that other studies present mixed results**. This balanced perspective is vital for accurately conveying the current state of the literature.
2. **We acknowledge the limitations** of our study in terms of the generalizability of the findings. We explicitly state that **additional evidence is necessary to substantiate the claims made regarding VRE**, ensuring that our conclusions are firmly grounded in the available data.
3. **Utilizing cautious phrases** such as "may suggest" and "could potentially contribute" could potentially contribute’ instead of making definitive statements. For instance, we have replaced "VRE improves cognitive function" with **"VRE may improve cognitive function,"** effectively communicating the tentative nature of our findings.

We believe these changes enhance the rigor and credibility of our discussion, providing a more accurate representation of the potential benefits of VRE while also emphasizing the need for further research in this area. Thank you for your constructive feedback, which has been instrumental in improving the quality and integrity of our manuscript.

**Reviewer Comment 14**

 If possible, the discussion could be strengthened by delving deeper into the mechanisms through which VRE might exert its effects on various outcomes.

**Response:** We appreciate your insightful comment regarding the potential mechanisms by which virtual reality exercises (VRE) may influence various outcomes. Your suggestion to deepen the exploration of these mechanisms in the discussion is highly valued.

**Action Taken:** In response to your feedback, we have significantly expanded the discussion section to offer a more comprehensive analysis of the mechanisms underlying the effects of VRE. The specific revisions are as follows.

1. **Discussion of Engagement and Motivation**: We elaborated on the role of VRE in enhancing participant engagement through immersive experiences, which can lead to increased motivation and adherence to exercise programs. For instance, we revised line 352 from "VRE can increase motivation" to "The immersive nature of VRE may lead to heightened motivation, which is essential for improving physical activity levels."
2. **Exploration of Cognitive Function**: We have included a detailed discussion on how VRE may facilitate cognitive engagement by requiring participants to interact with dynamic environments. In line 360, we modified the text from "VRE may help cognitive function" to "VRE potentially stimulates cognitive function by challenging participants to make quick decisions and adapt to changing virtual scenarios."
3. **Highlighting Social Interaction Effects**: We addressed the potential of VRE to promote social interaction and support, which could further enhance its effectiveness. In line 375, we revised the statement from "VRE can be done alone or with others" to "Engaging in VRE in a group setting may foster social bonds, providing psychological benefits that contribute to overall well-being."
4. **Consideration of Physiological Responses**: Finally, we examined the physiological responses associated with exercise in a virtual environment, positing that increased heart rate and caloric expenditure may arise from the interactive nature of VRE. We updated line 385 from "VRE involves physical activity" to "The interactive features of VRE may elicit greater physiological arousal, potentially leading to enhanced cardiovascular benefits."

**These enhancements are reflected in the discussion section, particularly between lines 350 and 390, where we now present a well-rounded perspective on the mechanisms through which VRE may exert its effects on various outcomes. Incorporating these insights significantly strengthens the discussion and provides a more thorough understanding of the potential benefits of VRE.**

 We are grateful for your valuable feedback, which has contributed greatly to the improvement of our manuscript **.**
